# Predicting Chiral Crystallization of Molecules Without Stereocenters

**DOI:** 10.3390/molecules31214013

**Published:** 2026-11-06

**Authors:** Lior Tobaly, Zion Elani, Yitzhak Mastai, David Avnir, Inbal Tuvi-Arad

**Affiliations:** 1 School of Business Administration, Bar Ilan University, Ramat-Gan 5290002, Israel; 2 Faculty of Engineering, Bar-Ilan University, Ramat Gan 5290002, Israel; 3 Department of Chemistry and The Institute of Nanotechnology, Bar-Ilan University, Ramat-Gan 5290002, Israel; zion.elani@biu.ac.il (Z.E.); mastai@biu.ac.il (Y.M.); 4 Institute of Chemistry and Center for Nanoscience and Nanotechnology, The Hebrew University of Jerusalem, Jerusalem 9190401, Israel; 5 Department of Natural Sciences, The Open University of Israel, Raanana 4353701, Israel

**Keywords:** chirality, Sohncke space groups, machine learning, explainable AI (XAI)

## Abstract

Given an achiral molecule which lacks a stereocenter, will it form chiral crystals? Predicting the crystallization symmetry mode of molecules has long been a central challenge in structural chemistry. Particularly enigmatic has been the observation that achiral molecules can give rise to chiral crystals, assuming any of the 65 Sohncke space groups. While case-specific interpretations have been proposed over the years, no general and reliable predictive model for that phenomenon has emerged. Here, we present an efficient solution to this long-standing challenge. We do so by developing a high-accuracy artificial intelligence-based framework, capable of predicting whether an organic molecule lacking stereocenters will form either chiral or achiral crystals. With a carefully curated dataset of organic molecules devoid of stereocenters, we trained a supervised neural network classifier to predict whether such molecules crystallize in Sohncke or non-Sohncke space groups, while identifying the molecular descriptor patterns that favor each outcome. The model achieved a receiver operating characteristic area under the curve (ROC-AUC) of 0.954 on a balanced held-out test set drawn from the same data source. Analysis of the descriptors that most influence the model’s predictions identifies molecular features that are statistically associated with each outcome. Predictions of the crystallization mode of anthracene and helicene derivatives exemplify these principles and are discussed in detail. Altogether, our predictive model opens new pathways for the rational design of molecules capable of forming chiral crystals and innovative avenues for crystal engineering, tailored for the many applications of chiral crystals, ranging from enantiomorphic drug safety considerations to enantioselective catalysis, and more.

## 1. Introduction

### 1.1. The Phenomenon of Chiral Crystallization of Achiral Molecules

**Figure 1:**
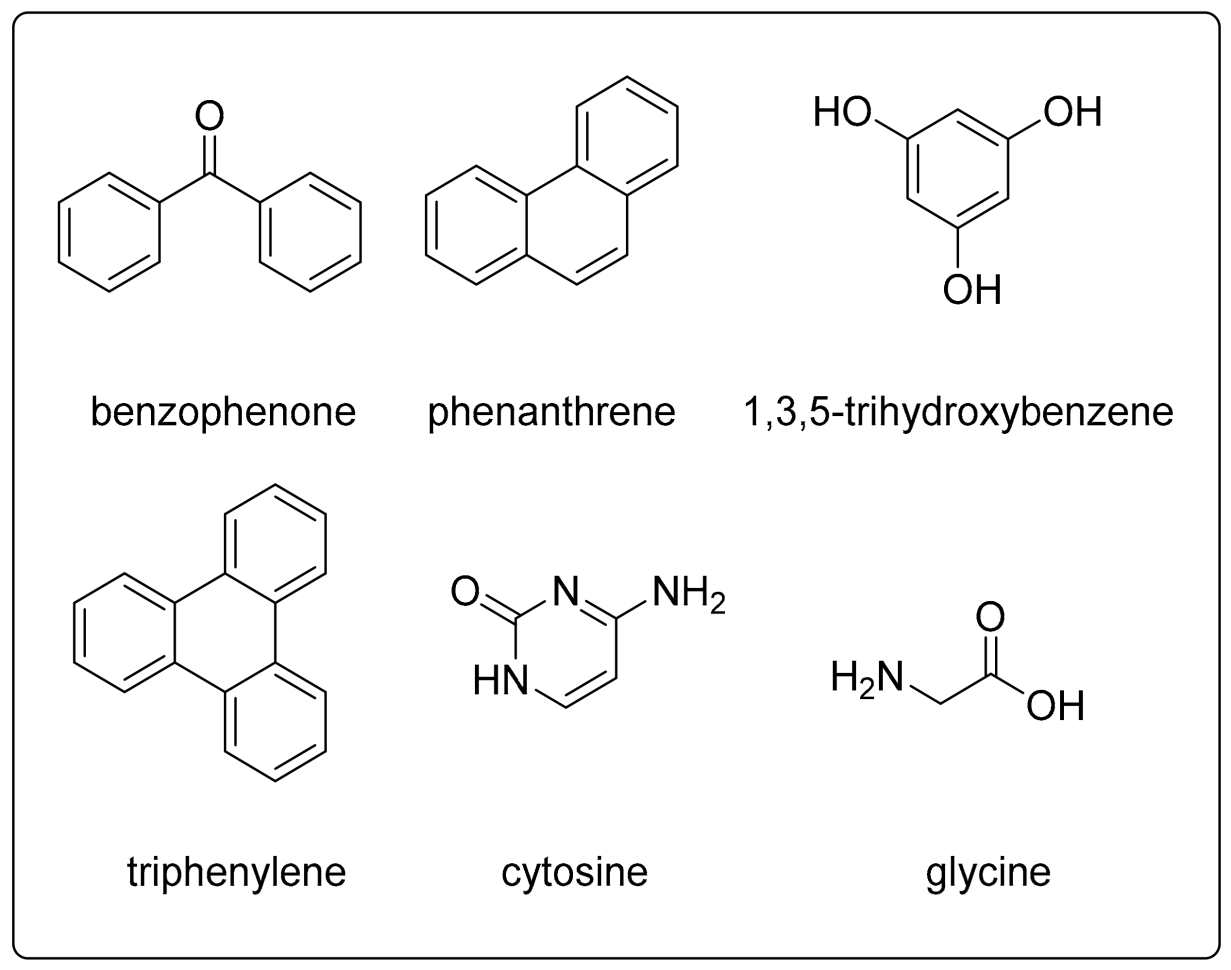
Examples of molecules that are achiral in solution but create chiral crystals.

A major challenge in the general study of crystallization has been to predict the non-trivial phenomenon of achiral molecules leading to chiral crystals. It is a spontaneous symmetry-breaking process by which achiral molecules end up upon crystallization as conglomerates of left- and right-handed crystals. While chiral molecules that crystallize enantiopurically always provide chiral crystal packing by adopting one of the 65 Sohncke space-group symmetries, it turns out that for achiral molecules, both options exist, providing either achiral or chiral crystals. It has been estimated that about 8% of all chiral crystals originate from achiral molecules [1]. For specific families of achiral molecules, the percentage is even higher, and an example is the simply substituted benzophenones, for which the percentage was estimated to be as high as 24% [1]. In fact, it is quite surprising that even the highly symmetric parent unsubstituted benzophenone crystallizes mainly in space-group symmetry *P*2_1_2_1_2_1_, which commonly hosts chiral crystals (Refcode = BPHENO22) [2]. Indeed, this symmetry-breaking phenomenon has always fascinated researchers, to the degree that many reports are devoted just to describing this phenomenon for specific molecules. Figure 1 presents additional examples of highly symmetric simple achiral molecules which lead to chiral crystals: phenanthrene (*P*2_1_, Refcode = PHENAN08) [3], 1,3,5-trihydroxybenzene (phloroglucinol, *P*2_1_2_1_2_1_, Refcode = PHGLOL01) [4], and triphenylene (*P*2_1_2_1_2_1_, Refcode = TRIPHE19) [5]. Some examples of achiral biomolecules that crystallize chirally are cytosine, which crystallizes in *P*2_1_2_1_2_1_ (Refcode = CYTSIN03) [6]; the only achiral proteinogenic amino acid glycine, the most stable polymorph of which (the gamma-polymorph) crystallizes in the space groups *P*3_1_ (#144, Refcode = DOLBIR46) [7] and *P*3_2_ (Refcode = DOLBIR) [8]; and many more [1,9].

Understanding the parameters that affect chiral crystallization of achiral molecules and predicting such crystallization have practical applications for enantiomorphic drug safety studies [10], enantioselective catalysis [11], optical applications [12], enantioselective separations [13] and more [14]. Most reports on such chiral crystallizations involve ad hoc interpretations, tailored for the specific reported cases. For instance, Sasaki et al. [15] attributed the induction of crystal chirality in several achiral ammonium carboxylates to differences in the numbers of methyl groups in their structures, that is, to specific steric effects forcing the molecules into a helical arrangement. The ability to form strong hydrogen bonds is often cited as a case-specific explanation [16]. For instance, in the case of chiral crystallization of the simple symmetric achiral phenol, it has been proposed that the triad of three hydrogen-bonded molecules forces the phenyl groups to slightly rotate out of plane [1]. Analyzing various achiral molecules, mainly biomolecules and drug molecules, Borchardt-Setter and Yu [10] suggested that a main parameter dictating the mode of crystallization is the energy gain: if it is larger for chiral than achiral crystallization, the former type of molecules occurs. In their classical review, Matsuura and Oshima [1] proposed “three important requirements for the generation of chirality from achiral organic compounds”: the rotational freedom of bonds, the ability to generate helicity, and the ability to form head-to-head columnar stacking. While applicable to specific cases described there, many chiral crystallizations do not follow these three rules. Other systematic approaches to understand this phenomenon rely on the synthesis and crystallization of a homologous series of achiral molecules in order to elucidate how small variations in structure might affect crystal symmetry. Examples include the study by Lennartson et al. [17] of simple diaryl ethers and the study by Ziach and Jurczak [18] of a library of macrocyclic imines. It has also been noticed that the formation of salt crystals from achiral cations and anions is a potential route of inducing chiral crystallinity. For example, Sasaki et al. [15] investigated the chiral crystallization of achiral arylamine/alkyl-carboxylate salts in the P1 and P2_1_ space groups and concluded that what dictates the nature of crystallization is a subtle balance between strong charge-assisted hydrogen bond interactions and weaker van der Waals interactions.

It is this subtleness of balance between so many specific parameters that renders the generalized prediction of chiral crystallization of achiral molecules so challenging. Relying on advanced computational AI methodologies, our study contributes an important step toward solving this challenge and provides an efficient, high-accuracy predictor of whether molecules lacking stereocenters crystallize in Sohncke or non-Sohncke space groups, while identifying a set of molecular descriptors that are associated with each crystallization outcome.

### 1.2. Computational Methods for Predicting Crystal Structures

Computational methods for predicting crystal structures and properties are rapidly advancing fields, with machine learning (ML) offering powerful tools to enhance and, in some cases, replace traditional methods. One branch of these methods is crystal structure prediction (CSP) simulations, which are based on density functional theory, specially designed force fields, and sophisticated optimization algorithms [19]. In this context, predicting the structure of organic crystals is considered very challenging due to the effects of weak intermolecular forces and molecular flexibility, which increase the complexity of the conformational landscape. While this approach is computationally expensive, the incorporation of ML algorithms can reduce the computational costs and increase prediction accuracy [20,21].

Applying ML methods to classify pair distribution functions (PDFs) offers a lower computational overhead. These functions rely on diffraction data and can characterize both crystalline and disordered materials by measuring the probability of finding atom pairs at a given distance, without assuming periodic atomic arrangements. As an example, the PDFitc set of AI-based tools developed by Yang and coworkers [22] provides online access for structure, space group, and similarity mining based on such data. The space-group predictor uses a pre-trained convolutional neural network model that estimates a list of the most likely space groups that match a given structure. Although effective, PDF-based methods rely on experimental measurements, limiting their use in the absence of such data.

A third approach to crystal structure prediction relies on machine learning models trained on 3D structural data extracted from large crystallographic databases such as the Cambridge Structural Database (CSD) [23], the Crystallographic Open Database (COD) [24], and others which contain extensive experimental measurements of known crystal structures. Once trained, these models can be applied to new systems without requiring experimental data, enabling rapid and computationally efficient predictions. This makes the approach not only a scalable alternative to traditional CSP methods but also an effective solution to the key limitation of PDF-based techniques, which depend on experimentally measured diffraction data for every prediction. By leveraging existing crystallographic datasets, these AI-driven models can efficiently screen candidate structures, estimate crystallization propensity, and identify symmetry or chirality even when no experimental information is available. One application of this approach is concerned with predicting the space groups of crystals given the material’s elemental composition [25,26,27]. Common ML algorithms for this task include Random Forest (RF) and Multilayer Perceptron (MLP) neural networks. Rather than providing a deterministic answer, such models usually return a ranked list of the most probable space groups for a given composition, reflecting the inherent complexity and ambiguity of crystallization behavior. Multilabel classifiers are also being developed to predict polymorphism [27], though this area still requires significantly more data for reliable predictions.

Prediction of chirality is another aspect, given its importance in drug discovery and crystal design [20,28]. Shimono et al. [29] developed a logistic regression model to classify the chirality of a dataset of binary inorganic compounds, with discrimination performance of 68.6%. According to this model, elements from groups 14 or 16, as well as groups 7, 8, 10, and 12 (transition metals), tend to increase the probability of forming chiral crystals. Identifying chirality in organic crystals often requires manual curation, since key metadata are frequently missing in the deposition of structures in databases like the CSD [23]. Walsh et al. [28] showed that despite the deposition of over 108,000 new crystal structures in the CSD between 2020 and 2021, none were identified as spontaneously resolving conglomerate crystals in their CIF file within that period, highlighting a lack of systematic recording of this behavior. On the other hand, manual inspection identified 343 new chiral materials exhibiting this behavior. While crystallographic databases are crucial resources [25,28,30], these findings point to a need for better communication and standardized data reporting between synthetic and crystallographic communities [28]. Due to its central role in chemistry, developing accurate ML models for chirality prediction remains a key objective, particularly for models capable of extending their applicability across diverse branches of molecular structure.

It is seen from this brief literature review that none of the existing approaches provide an efficient solution to the challenge of predicting the chiral/achiral outcome of the crystallization process. Here, we present **CrysChiP** (**Crys**tal **Chi**rality **P**redictor), an AI-driven framework that integrates advanced feature engineering, deep learning, and explainable AI tailored to predict whether organic molecules lacking stereocenters will create chiral or achiral crystals upon crystallization.

## 2. Results and Discussion

### 2.1. CrysChiP: Model Construction

CrysChiP was developed using a dataset comprising tens of thousands of crystallographic entries from the CSD. Various resampling strategies were employed to create a balanced dataset for model training and to mitigate data scarcity and distribution imbalance across the 230 space-group symmetries. Entries were restricted to crystals of organic molecules lacking R/S-assignable stereocenters, as identified using the CSD Python API (see the Methodology Section 3.1.), each composed of a single type of molecular component with up to 100 atoms without stereocenters. A total of 106,756 crystals for which a SMILES, (Simplified Molecular Input Line Entry System) string can be defined, were retrieved, forming the source dataset. Chiral crystals constitute 10.6% of the source dataset. To ensure that the model effectively learns to identify molecules likely to form chiral crystals, we derived a training set (11,200 structures) and a held-out test set (4800 structures) from the source dataset, each with a 50:50 balance of chiral crystals (in Sohncke space groups) and achiral crystals (non-Sohncke space groups).

A major contribution of the framework is the incorporation of the SHapley Additive exPlanations (SHAP) method [31], which provides global feature importance and local directional insights into how specific molecular descriptors drive the prediction of crystallization outcome. The model demonstrates strong performance across a variety of scoring functions, while maintaining interpretability and intuitive usage. Overall, CrysChiP advances the application of deep learning and explainable AI in crystallography, offering a path to extend our understanding and extract meaningful molecular insights on crystallization outcomes. Our model, CrysChiP, achieved high accuracy across multiple scoring metrics and identified a compact set of molecular descriptors, revealing that certain descriptors consistently characterize chiral crystals while others are associated with achiral outcomes. Detailed model performance and analyses are presented below. To improve the flow of the main text, methodological details are presented at the end of this paper and elaborated further in the Supplementary Materials (SM).

### 2.2. Model Performance

Using the SMILES strings as input, 2563 non-redundant two-dimensional (2D) molecular descriptors (commonly called features) were calculated for each structure. The K-best algorithm was used to select the K most influential features (see the Methodology Section 3.2.). To select a representative model, we sought a compact descriptor set that balances predictive performance with chemical interpretability. Model performance, as described below, was tested for several values of K ∈ {5, 6, 7, 10, 12, 20, 50} as well as the maximum K with all 2563 descriptors.

The models were assessed using a variety of scoring functions presented in Table 1: Accuracy, Macro-F1, ROC-AUC, and PR-AUC (see the Methodology Section 3.4.). Accuracy and Macro-F1 are defined as composite formulas of four quantities, TP (True Positive), FP (False Positive), TN (True Negative), and FN (False Negative), whereas ROC-AUC and PR-AUC evaluate model performance across decision thresholds. Herein, positive/negative refer to chiral/achiral crystals, while true/false correspond to correct/incorrect prediction of the crystal’s chirality, respectively. In simple terms, Accuracy is the fraction of molecules predicted correctly overall; Macro-F1 reflects how well a model performs across both chiral and achiral classes by giving each class an equal contribution to the final score, regardless of how common or rare it is; ROC-AUC summarizes how well the model separates the two classes across all decision thresholds; and the PR-AUC score summarizes how well a model distinguishes the positive class across different decision thresholds, placing particular emphasis on how reliably the model identifies positive cases when they are relatively rare. The metric formulas are provided in Table S1 of the SM.

As is evident from Table 1, for K > 6, the models exhibit high predictive quality, with ROC-AUC and PR-AUC > 0.94, demonstrating strong discrimination across decision thresholds, while rounded Accuracy and Macro-F1 range from 0.859 to 0.890 at the selected operating thresholds, implying that the overall strategy is effective. For the top-ranked configurations (K ≥ 6), K-Best forms a nested feature set: a larger K primarily adds descriptors on top of those already selected at a smaller K. While performance generally improves with K, the incremental gain becomes marginal beyond the mid-range, whereas the explanation length grows linearly with K. In parallel, the distribution of descriptor families stabilizes already at K = 7 and becomes increasingly redundant at K ≥ 20, suggesting that larger K mainly reinforces already-captured feature types rather than adding qualitatively new explanatory factors. We selected K = 12 as a representative compromise between predictive performance and a manageable explanation, rather than as a formally established knee-point or the most accurate configuration. Relative to K = 50, it uses 38 fewer descriptors with a 1.42-percentage-point reduction in held-out accuracy (87.56% versus 88.98%). This choice was informed by the held-out comparisons, rather than by cross-validation-based selection.

To complement these performance metrics, we also examined prediction errors at the selected operating threshold using confusion matrices. For this purpose, we calculated the confusion matrix of each model on the held-out test set to classify predictions as chiral or achiral (see Table S2 in the SM). The confusion matrix graphically summarizes classification performance by comparing the actual class (*Y*-axis) with the predicted class (*X*-axis), with correct classifications appearing along the diagonal and misclassifications in the off-diagonal cells. This visualization provides a clear view of how each model balances FP (predicting a chiral crystallization outcome when the actual crystal is achiral) and FN (predicting an achiral crystallization outcome when the actual crystal is chiral). In a screening application aimed at identifying candidates for chiral crystallization, FN may be of particular practical concern because potentially relevant candidates would be excluded from further consideration. Missed chiral candidates can translate into an irreversible loss of rare high-value structures and missed opportunities for downstream characterization. We therefore considered FN rates as an additional practical criterion when selecting the representative model. Compared with K = 10, K = 12 reduces FN from 405 to 302 (16.9% to 12.6% of Sohncke-class crystals), while FP increases from 247 to 295; the net gain is 55 correct classifications. Relative to K = 2563 (Max), K = 12 reduces FN from 340 to 302 but increases FP from 202 to 295, giving 55 fewer correct classifications overall. Thus, K = 12 represents an interpretability–performance compromise, not an overall improvement on the full-feature baseline. These comparisons are descriptive because the held-out results informed the representative-model choice.

**Figure 2:**
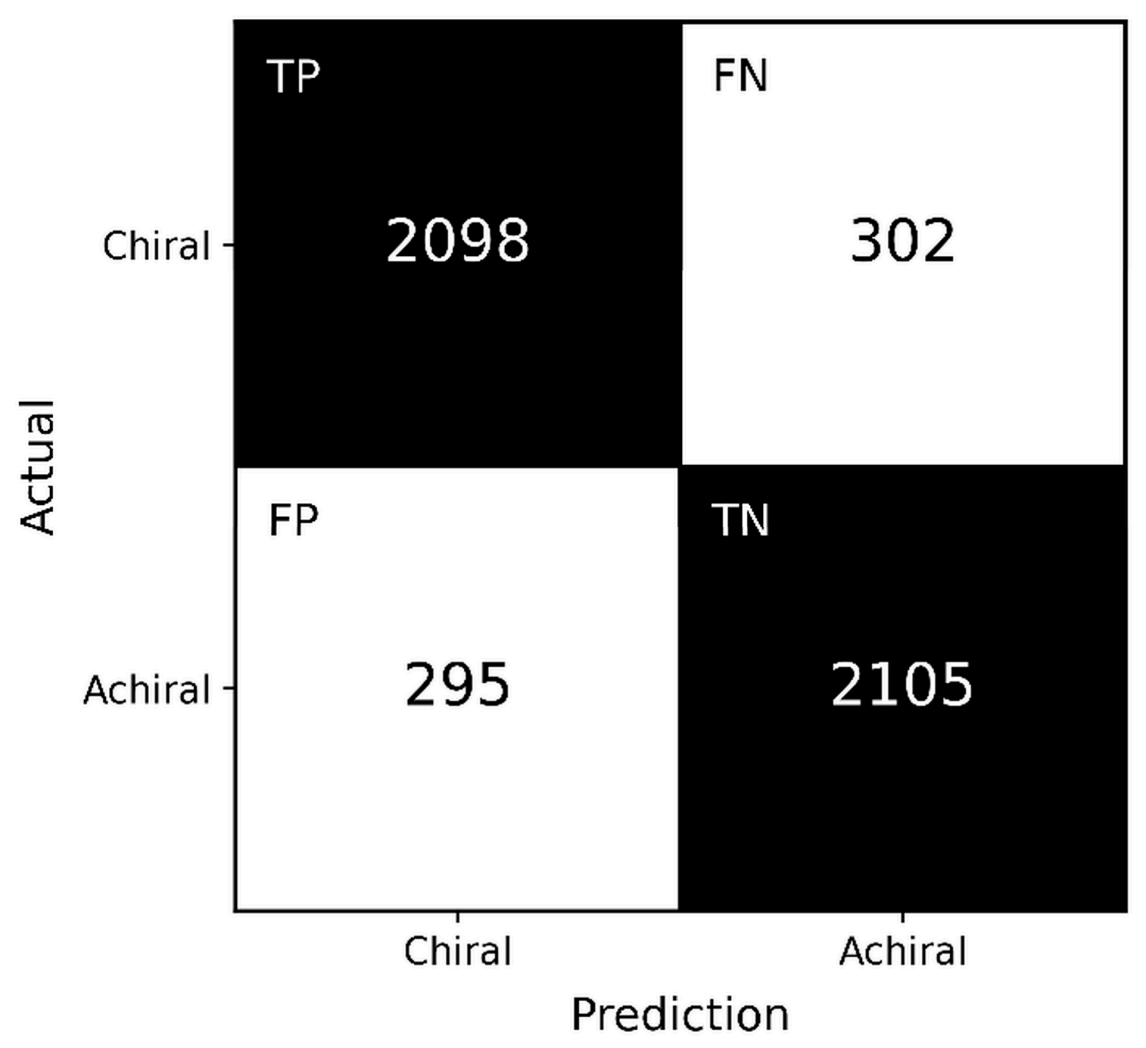
Confusion matrix of 4800 molecules in our held-out test set for CrysChiP (K = 12).

Figure 2 presents the confusion matrix of CrysChiP. Among the 2400 molecules lacking a stereocenter that form crystals in Sohncke space groups, the model correctly predicted a chiral crystallization outcome for 2098 molecules (TP) and incorrectly predicted an achiral crystallization outcome for 302 molecules (FN). Conversely, among the 2400 molecules that crystallize in non-Sohncke space groups, the model correctly predicted an achiral crystallization outcome for 2105 molecules (TN) and incorrectly predicted a chiral crystallization outcome for 295 molecules (FP).

Based on the classification rates observed in the balanced held-out test set, the sensitivity is 2098/2400 = 87.4% and the specificity is 2105/2400 = 87.7%. For illustration, if these class-conditional rates remain unchanged at a Sohncke-class prevalence of 10.6%, the projected positive predictive value (PPV) and negative predictive value (NPV) are approximately 45.7% and 98.3%, respectively. These projections follow PPV = Se × π/[Se × π + (1 − Sp) × (1 − π)] and NPV = Sp × (1 − π)/[(1 − Se) × π + Sp × (1 − π)], where Se is sensitivity, Sp is specificity, and π is the positive-class prevalence. Thus, a positive prediction would enrich the source prevalence approximately 4.3-fold, while about 54.3% of positive predictions would still be false positives. These are prevalence-adjusted projections, not measurements on an external or naturally distributed held-out test set. The 10.6% prevalence describes the retrieved CSD source dataset and need not represent other chemical populations or screening settings.

**Figure 3:**
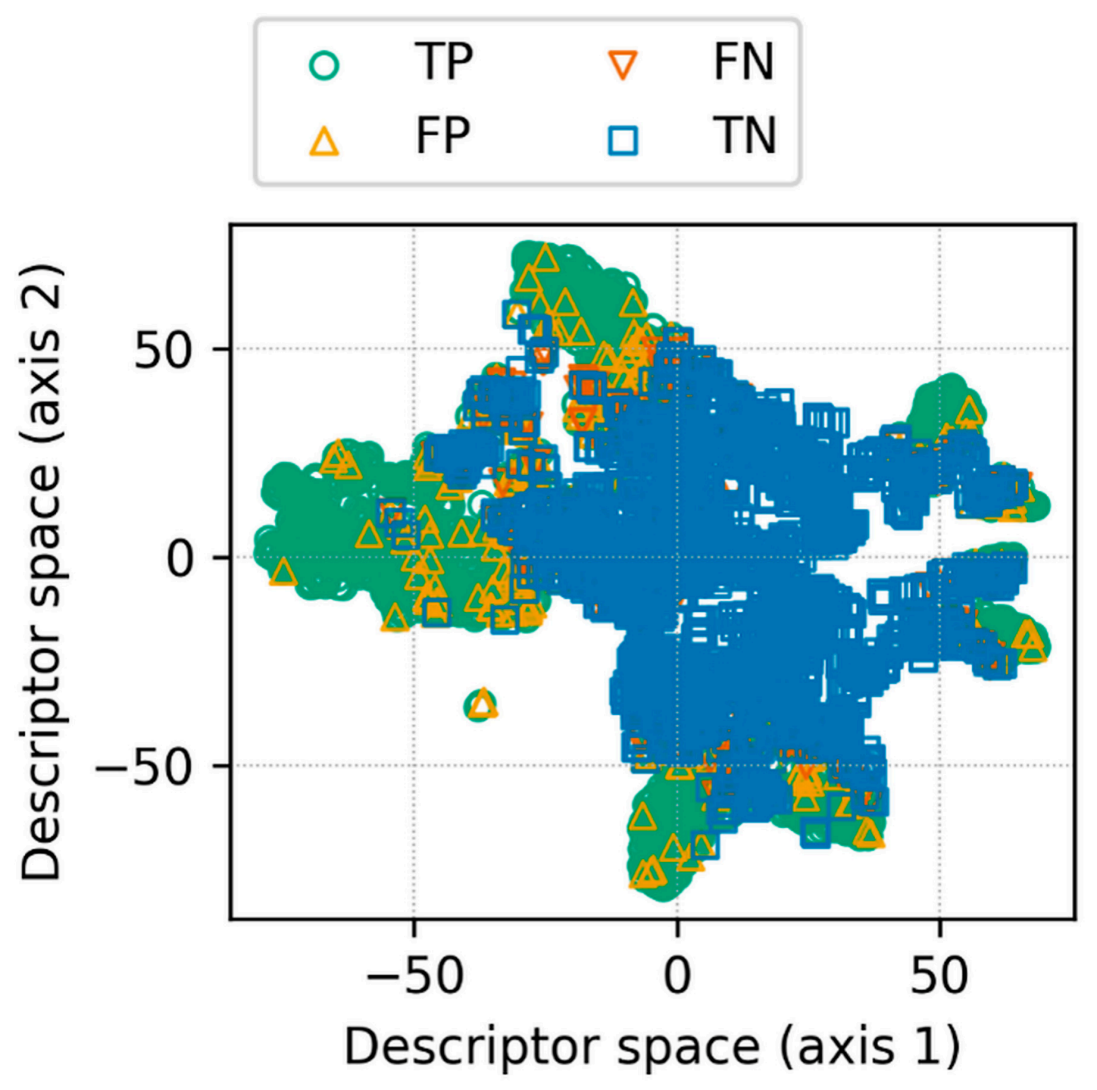
t-SNE in descriptor space; see text for full explanation and interpretation.

Another common way to analyze the performance of machine learning models is the t-Distributed Stochastic Neighbor Embedding (t-SNE) method, which reduces high-dimensional data into a low-dimensional space. In our case, it projects the 12 features used by CrysChiP into a 2D representation shown in Figure 3. The main advantage of t-SNE is its ability to visualize similarity relationships: molecules with similar feature profiles tend to cluster together in the map, while molecules with distinct characteristics appear in separate regions. Figure 3 indeed shows a pronounced single achiral-enriched region (TN predictions, blue) surrounded by several chiral-enriched regions (TP predictions, green), with the FP and FN predictions mainly at the interface between the chiral and achiral classes. An interpretation of this observation is as follows: The blue central region of molecules that form achiral crystals belong to a broad but fairly homogeneous class of molecules. The green TP regions—the focus of our report—display a distinctly different pattern: several smaller clusters of chiral crystals formed by molecules without stereocenters, appear around and outside the achiral core. These results suggest that descriptor combinations associated with chiral crystallization are more heterogeneous than those associated with achiral crystallization and cannot be characterized by a single unified explanation, which is consistent with the major conclusion of our study. Nevertheless, the presence of several distinct clusters suggests that chiral crystallization is associated with particular combinations of molecular properties, including the ability to create hydrogen bonds, conformational flexibility, steric hindrance and more, and not from endless possibilities of these combinations. Finally, the FP and FN cases lie mainly at the interfaces between the TN- and TP-enriched regions, indicating areas of descriptor space in which the two crystallization outcomes are less clearly separated.

To summarize so far, CrysChiP performance was tested on a balanced held-out test set of 4800 molecules without stereocenters, where 2400 form chiral crystals and 2400 form achiral crystals. This design supports comparison of performance across the two classes, but the reported Accuracy, Macro-F1, and PR-AUC describe the balanced evaluation distribution and should not be assumed to hold under other class distributions. Performance on this held-out test set is strong (Accuracy = 0.876, Macro-F1 = 0.876, ROC-AUC = 0.954, PR-AUC (chiral) = 0.956), far above random baselines. Overall, these embeddings and metrics show that the compact, deployable descriptor subset (K = 12) of CrysChiP captures a robust structure–outcome signal, motivating a closer look at the chemical meaning of the features that drive this performance. For additional analyses of all our models, including threshold-dependent behavior, cost-sensitive analysis, prediction stability, calibration, and confidence-based selectivity, see the SM (Figures S1–S9, Tables S3–S7).

### 2.3. The Descriptors

Table 2 presents the 12 descriptors (features) that construct CrysChiP with short descriptions. These are related to various properties of the molecules, including the following: atomic composition, which captures the diversity of atom types (TIC0) and the environment around them (ZMIC3); electronic properties, which capture the distribution of charge by means of the ionization potential (ATSC0i), bond polarizability (bpol) and topological charge (GGl1); weighted graph–spectral descriptors, which encode the combined effect of topological connectivity and atomic properties such as mass distribution (SM1_Dzm), intrinsic state (BCUTs-1h) and atomic numbers (BCUTZ-1h); structural complexity in terms of the number of structural fragments (fragCpx) and the level of branching (mZagreb1); and topological connectivity, which describes the local spatial arrangement (Xc-3d) and the global shape and electronic distribution (ETA_eta). A thorough description of each descriptor can be found in Todeschini and Consonni [32].

To interpret the chemical logic underlying CrysChiP, we calculated SHAP [31] values for each descriptor for all the molecules in the held-out test set (see the Methodology Section 3.4.). SHAP values quantify how each feature contributes to a model’s prediction by showing the magnitude of its effect and its directional influence—positive (prediction of a chiral crystallization outcome) or negative (an achiral crystallization outcome). These can provide quantitative information about feature importance: both locally (for each molecule) and globally (summarized across the entire dataset). For each of the 12 features of CrysChiP, the SHAP values vary from molecule to molecule, making the relative local importance of the features sample-dependent. Because chiral and achiral predictions drive SHAP values in opposite directions, the mean SHAP value per descriptor across the entire held-out test set is not very informative. However, analyzing the TP and TN subsets separately provides a much clearer picture. In what follows we apply this approach to highlight the global contribution of each feature, the sample-specific dominant descriptors and the SHAP rank trends that examine how influence is distributed across features.

The mean absolute SHAP values quantify the importance of each feature in the TP and TN subsets. For the TP subset, five out of twelve features show the highest mean absolute SHAP values, and together account for 73% of the overall feature contribution to the model’s correct classification: ZMIC3 (22%), SM1_Dzm (19%), BCUTZ-1h (14%), bpol (9%), and TIC0 (9%). We refer to these descriptors as the TP-dominant set. Chemically, these descriptors capture aspects of the molecule’s mass distribution, electronic topography, bond polarizability, and atom type diversity. A plausible explanation is that uneven distribution of these properties can introduce directionality in the molecular structure, which may favor the formation of chiral crystals. For example, high SM1_Dzm reflects features of the molecular graph weighted by atomic mass, while high bpol values indicate greater differences in atomic polarizabilities across bonds. Both may reflect less uniform distributions of molecular properties that could favor lower-symmetry crystal packing.

For TN predictions, six out of twelve features account for 71% of the overall influence on the model’s decision: ATSC0i (21%), SM1_Dzm (13%), bpol (12%), ETA_eta (10%), Xc-3d (8%), and ZMIC3 (8%). We refer to these descriptors as the TN-dominant set. These descriptors capture the molecule’s ionization potential, mass distribution, bond polarizability, electronic distribution, and branching level. We note that ZMIC3, SM1_Dzm, and bpol appear in both groups, indicating that these descriptors are informative for distinguishing the propensity of molecules to form either chiral or achiral crystals.

**Figure 4:**
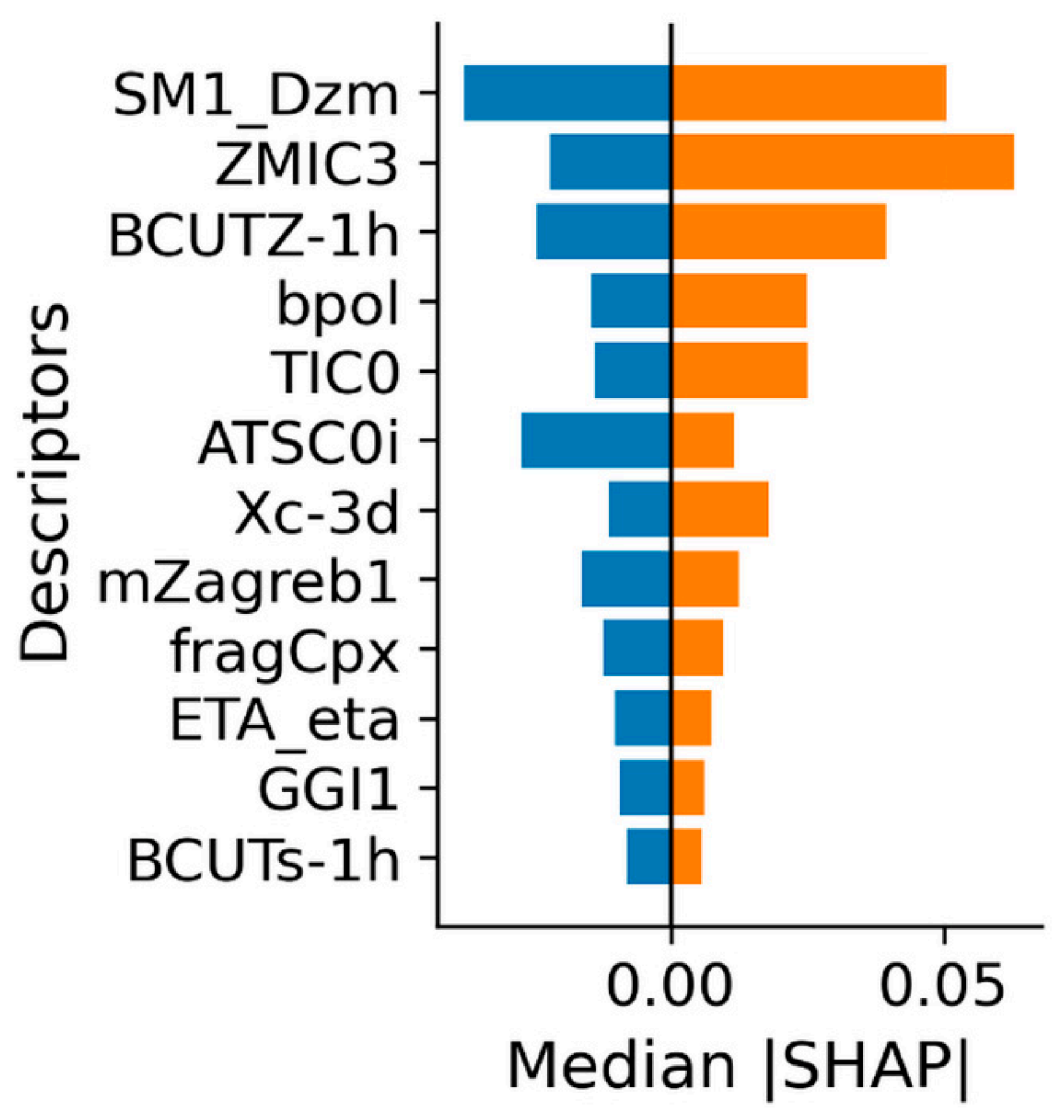
A tornado plot of the median absolute SHAP values. Blue—TN prediction; orange—TP predictions. Bar direction is used only to distinguish between the two classes.

To summarize the global importance, Figure 4 presents a tornado plot of the median absolute SHAP values of each feature, with blue and orange bars representing, respectively, the global contribution of each feature to the achiral and chiral predictions. The total bar length (i.e., the sum of the TP and TN median absolute values per feature) reflects the overall feature importance.

**Figure 5:**
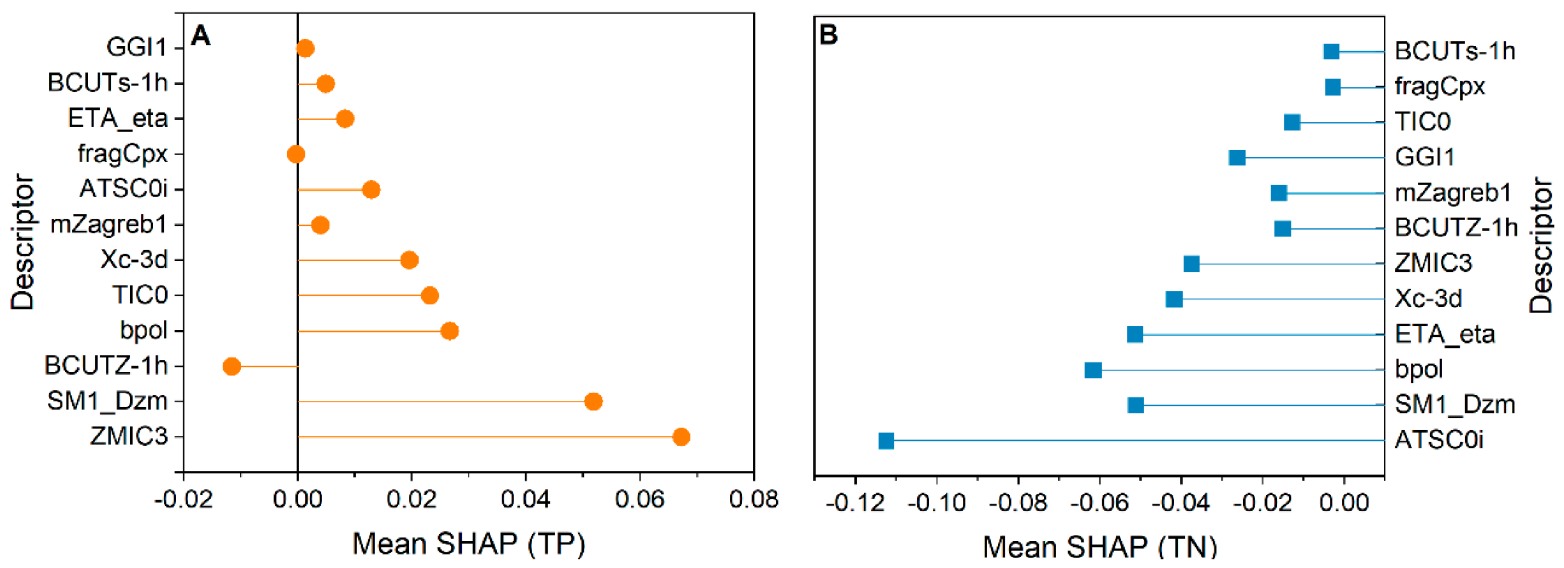
Mean SHAP values. (**A**) TP dataset; (**B**) TN dataset.

Figure 5A,B present the mean SHAP values of each feature for the TP and TN datasets, respectively, without taking the absolute value. Here, the SHAP values indicate the direction of the model’s prediction towards chiral and achiral crystallization outcomes. Note that the order of descriptors along the vertical axes in Figure 5 differs for the two plots, as these are sorted by their mean SHAP values. As discussed above, the TP-dominant set of descriptors is the most influential on TP predictions (Figure 5A). Most descriptors show positive mean SHAP values, indicating that they generally push the prediction toward a chiral crystal. In contrast, BCUTZ-1h, reflecting connectivity patterns influenced by heavy atoms and their electronic environments, exhibits a negative mean SHAP value. This suggests that CrysChiP interprets the levels of this descriptor in the held-out test set as generally inconsistent with chirality. For TP predictions, the positive contributions from other descriptors outweigh this negative influence, allowing CrysChiP to correctly classify the sample. For TN predictions (Figure 5B), the TN-dominant set of descriptors displays the most meaningful pattern of descriptor contributions. All descriptors show negative mean SHAP values, pushing the prediction toward the achiral class. Notably, the mean SHAP value of BCUTZ-1h is similar for both chiral and achiral predictions. This indicates that molecules in the held-out test set share certain connectivity signatures that the model consistently associates with achiral structures. As a result, BCUTZ-1h functions as a global counter-signal against chirality rather than a class-specific discriminator. It was included in the TP-dominant set due to the weight of its absolute contribution to the model decision for TP predictions (14%).

**Figure 6:**
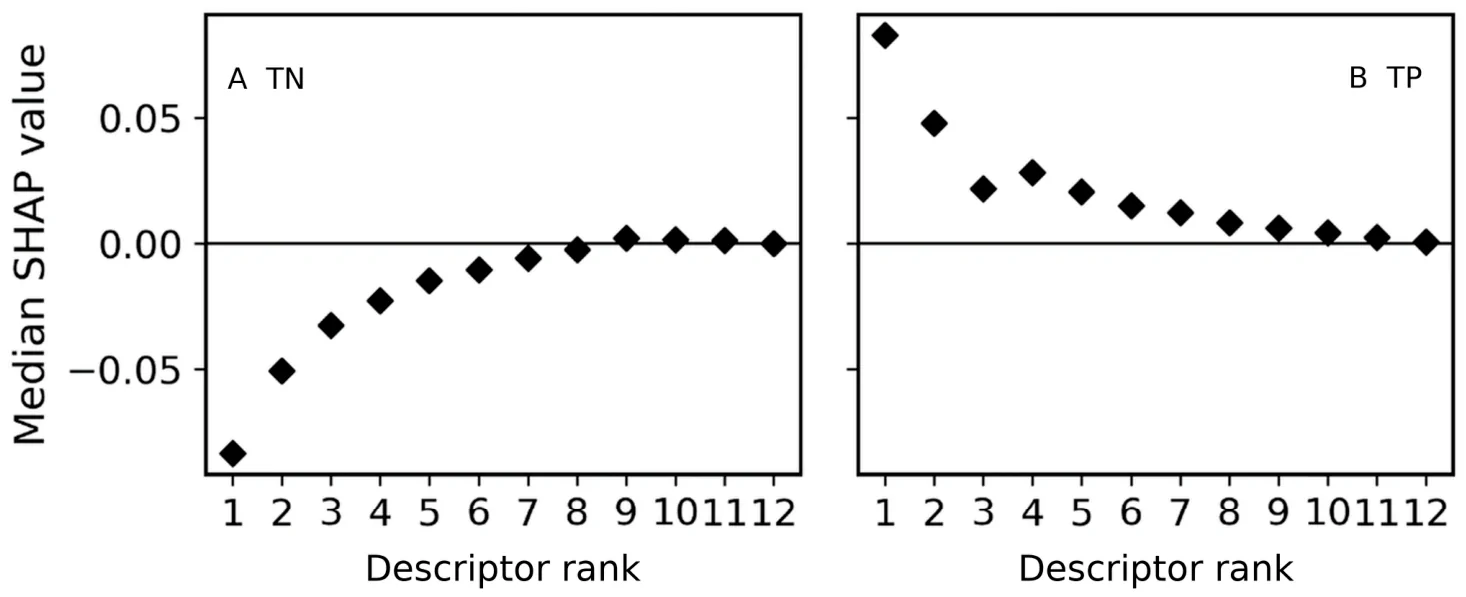
The trend of the median SHAP values for (**A**) TN and (**B**) TP predictions as a function of descriptor rank.

Beyond the most dominant features, it is informative to examine the trend of SHAP values to understand the sequence of contributions that the model relies on when producing correct predictions. Figure 6 displays the median SHAP values as a function of descriptor rank, calculated separately for TN and TP predictions. A rank of 1 corresponds to the feature with the largest absolute SHAP value for a given prediction, regardless of which descriptor set it belongs to. A rank of 2 corresponds to the feature with the second-largest absolute SHAP value, and so on. The trends extend and strengthen the above findings, showing that the same directional pattern persists across all ranked descriptors. The decrease in ranked median SHAP values indicates that the top five to six descriptors provide the core contribution to the model’s decision in each case. The remaining descriptors still contribute, but their role is more supportive: they refine, strengthen, and stabilize the final classification rather than driving it. TN predictions exhibit negative median SHAP values across the feature ranks, whereas TP predictions show positive medians, revealing a consistent class-specific trend. Overall, the patterns in Figure 6 show how CrysChiP is making decisions. To better understand the model, in what follows we analyze two specific examples.

Finally, we comment on the lesser extent misclassification. For 70% of the FN cases (chiral crystals incorrectly predicted as achiral), one of four features appears as the top-ranked feature in the SHAP analysis: SM1_Dzm (35%), BCUTZ-1h (17%), ZMIC3 (9%), and bpol (9%). Two additional features, ATSC0i and TIC0, each appear as the top feature in 8% of cases. Altogether, six features account for 86% of the false negative explanations. For FP cases (achiral crystals incorrectly predicted as chiral), three features dominate, BCUTZ-1h (31%), SM1_Dzm (29%), and ZMIC3 (19%), together explaining 79% of the cases. In both error types (FN and FP), the leading features come from both the chiral and achiral descriptor sets. This overlap likely contributes to the observed misclassification.

### 2.4. Examples of Predictions

**Figure 7:**
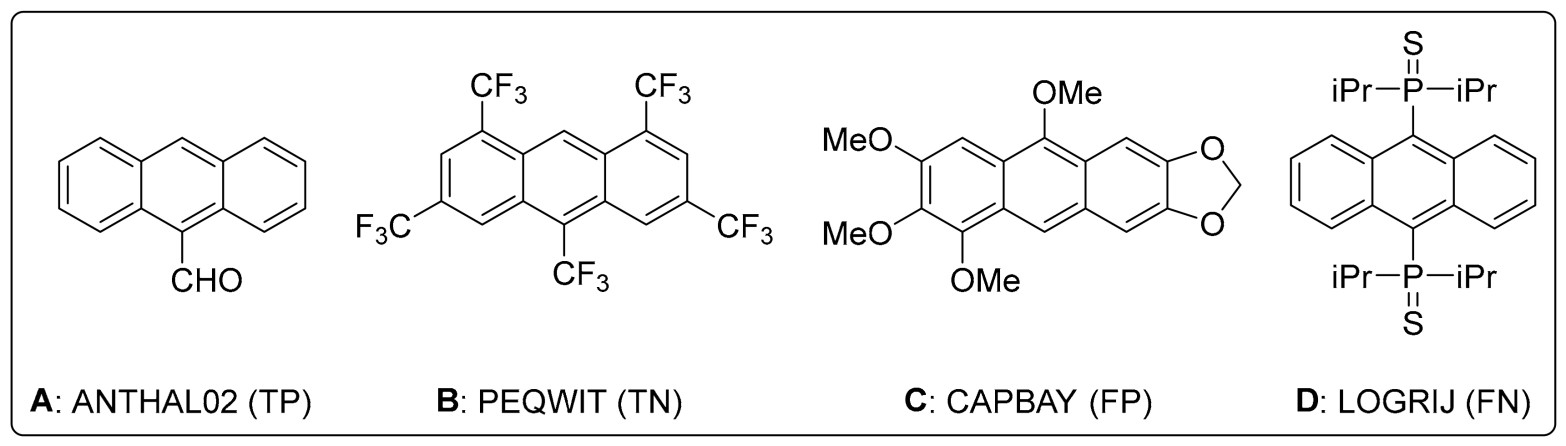
Four derivatives of anthracene. Space groups are listed in parentheses: (**A**) TP (*P*2_1_2_1_2_1_), (**B**) TN ($P\bar{1}$), (**C**) FP (*P*2_1_/c), and (**D**) FN (*P*2_1_2_1_2_1_).

**Figure 8:**
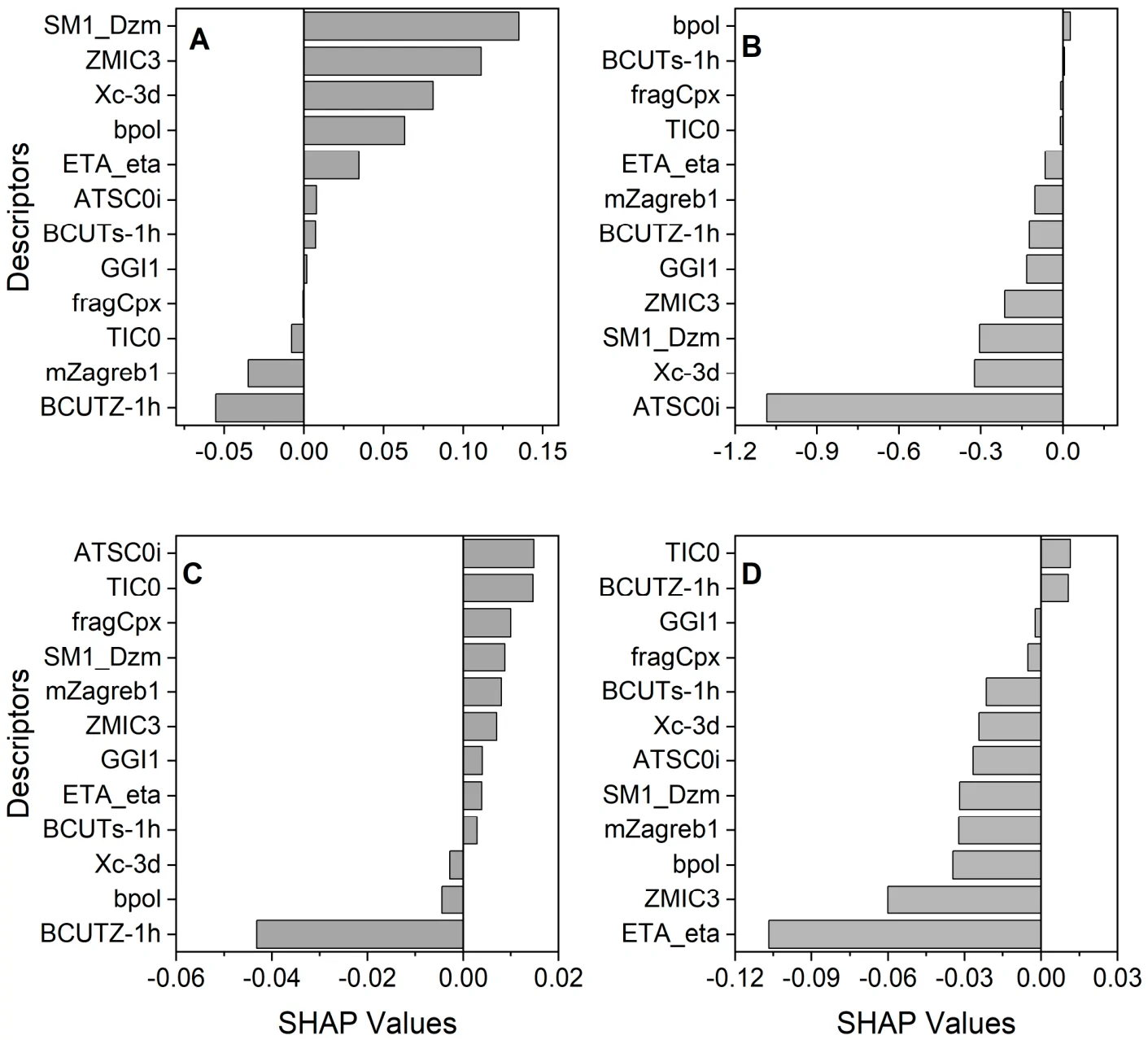
A set of tornado plots showing SHAP values per descriptor for four anthracene derivatives shown in Figure 7. (**A**) TP (ANTHAL02), (**B**) TN (PEQWIT), (**C**) FP (CAPBAY), and (**D**) FN (LOGRIJ).

Anthracene derivatives: Figure 7 displays four derivatives of anthracene from our held-out test set which share the same aromatic core structure. Two are correctly predicted to form chiral (Figure 7A: TP) and achiral (Figure 7B: TN) crystals. As it is constructive to examine the minority of the false predictions, the two others are an achiral crystal predicted to be chiral (Figure 7C: FP) and a chiral crystal predicted to be achiral (Figure 7D: FN). The corresponding SHAP values are shown as tornado plots in Figure 8A–D. Each plot is sorted according to the descriptor ranking for that specific molecule, which is why the order of features on the *Y*-axis differs between plots. Note also that the *X*-axis scales vary. These molecule-specific rankings reflect the individualized feature importance patterns captured by CrysChiP. Let us now propose a rationalization of these predictions.

Anthracene-9-carbaldehyde (ANTHAL02) crystallizes in *P*2_1_2_1_2_1_ (Figure 7A). The source of chirality here is a rotation of the aldehyde group out of the anthracene plane, either clockwise or counterclockwise. This could result from relief of steric hindrance, yet this requires further testing. On the other hand, 1,3,6,8,10-pentakis(trifluoromethyl)anthracene (PEQWIT) crystallizes achirally in $P\bar{1}$ (Figure 7B). We recall that Z = 2 with inversion symmetry $(P\bar{1}$) is a common crystallization mode, a phenomenon which has received intensive attention. In brief, it is related to the Kitaigorodskii’s close-packing argument, and to the fact that, except for *P*1, this space group is the only one which does not impose geometric constraints on molecular shape upon crystallization [17].

CrysChiP successfully predicted the chirality/achirality of the crystal for these two cases, classifying ANTHAL02 as TP and PEQWIT as TN. The accompanying different sets of descriptors presented in Figure 8A,B indeed relate to these modes of crystallization and to the intuitive explanation detailed above, as follows. ANTHALA02 (Figure 8A) shows mainly positive SHAP values while PEQWIT (Figure 8B) is mostly negative, in line with the general trends in Figure 5 and Figure 6. Comparing Figure 8A,B it can be seen that two descriptors create major differences and are part of the TN-dominant set: ATSC0i, which measures how the ionization potential is distributed across the molecular graph and characterizes the electronic topology of the structures, and Xc-3d, which describes the branching and connectivity of three-bond clusters in the molecular structure. Two other descriptors appear in both sets: ZMIC3, which quantifies the diversity of an atom’s neighborhood up to three bonds away, and SM1_Dzm, which is sensitive to asymmetric distribution of heavy atoms in the molecular structure. Both display significantly different SHAP values for ANTHAL02 and PEQWIT, with a sign that is consistent with the general trend presented in Figure 5: positive for TP predictions and negative for TN predictions.

Looking at the structures of the two molecules in Figure 7A,B, PEQWIT displays more even mass and charge distributions compared with ANTHAL02. The fact that the aldehyde group in ANTHAL02 rotates out of the symmetry plane is expressed by the descriptors as a less even and less symmetric distribution of charge and mass. The model has learned to associate such trends with Sohncke outcomes. Whether this association reflects directional packing within the unit cell remains to be tested.

Figure 7C,D display two other anthracene derivatives for which CrysChiP fails to provide correct predictions. CAPBAY (Figure 7C, FP) creates an achiral crystal in the *P*2_1_/c space group and is incorrectly predicted to be chiral by CrysChiP. Figure 8C shows that most of the SHAP values are positive, which may be related to the uneven mass and charge distribution in this structure (Figure 7C) and can push the model towards a chiral prediction. However, the prediction probability, *p* = 0.539, is only slightly above the decision threshold of 0.5 (see the Methodology Section 3.4.) and the positive SHAP values are generally small.

The case of LOGRIJ (Figure 7D) is another example of a wrong prediction. The crystal was refined in *P*2_1_2_1_2_1_; however, CrysChiP predicts that an achiral crystal will be formed, making it a FN case. The corresponding SHAP values in Figure 8D are mostly negative, as expected from achiral predictions, and this may be explained by the molecular symmetry: its 3D structure belongs to the *C*_s_ point group, whereas its 2D structure (the only input to the model) may look as if the molecule has higher symmetry. The most negative SHAP value in Figure 8D is of ETA_eta, which is part of the TN-dominant set. This descriptor captures the overall electronic shape. It accounts for core electrons, valence electrons, and lone pairs while comparing the molecule to a reference graph. Values closer to a symmetric reference may push the prediction toward the achiral class and, in this case, contribute to misclassification. Here, the prediction probability is *p* = 0.054, far below the decision threshold of 0.5.

Helicene derivatives: The training of CrysChiP was based on molecules that lack stereocenters. Molecules with other types of chirality such as helical and axial chirality were naturally part of the training set, but no special indications were given to help the model discriminate them from all other molecules. It was therefore encouraging to find that CrysChiP can successfully predict the crystallization mode of helicene derivatives with high accuracy. Table 3 presents the results for a set of helicene derivatives from the held-out test set, showing that the mode of crystallization of 21 out of 24 samples was predicted correctly. The top-ranked feature that directed most of the TP predictions is SM1_Dzm, which is included in both the chiral and achiral descriptor sets. For TN cases, three other features were found at the top: two from the TN-dominant set (ATSC0i and ETA_eta) and one from the TP-dominant set (BCUTZ-1h).

**Figure 9:**
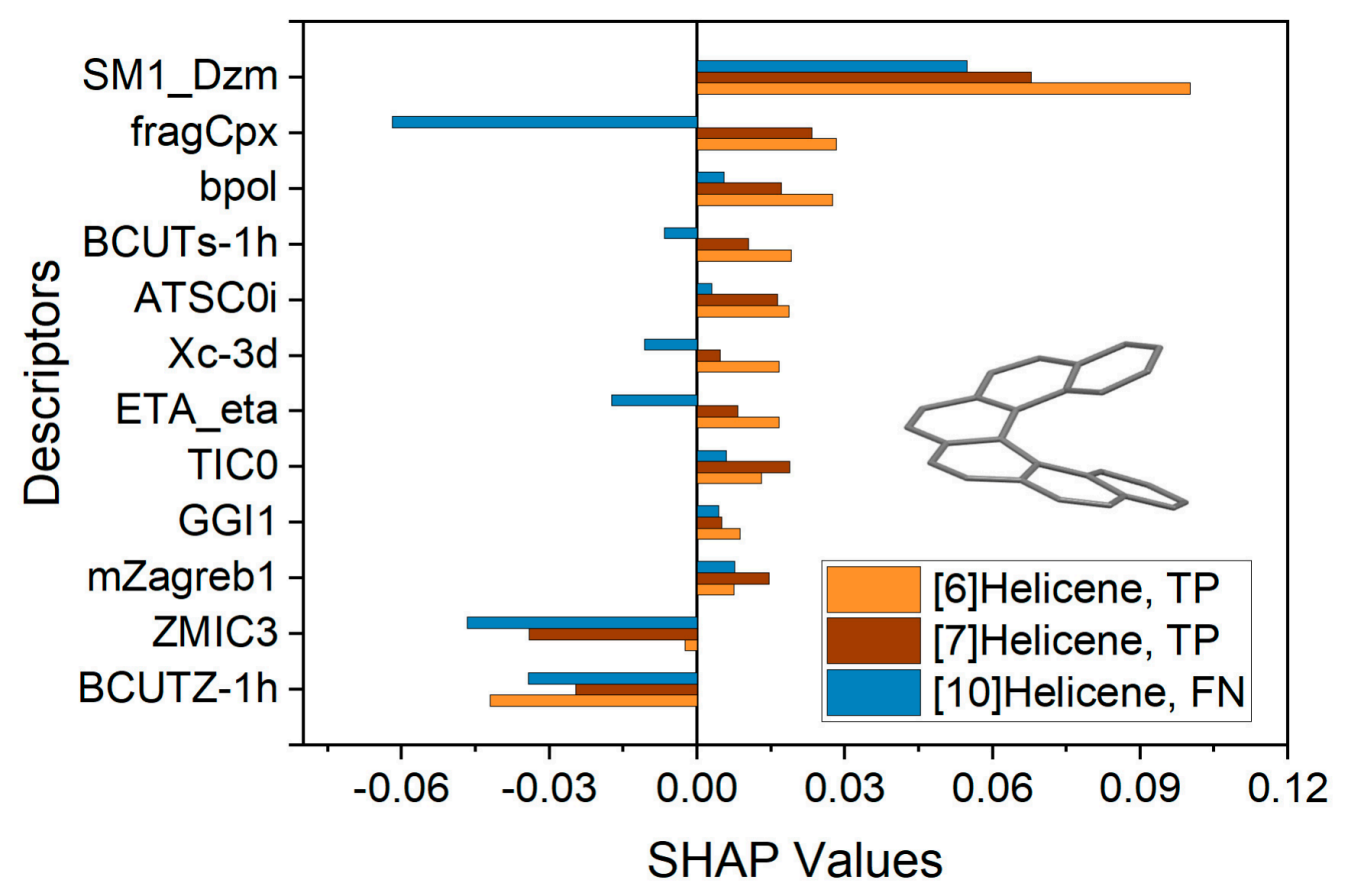
Tornado plot of SHAP values of CrysChiP descriptors for [6] (shown) and [7,10] helicene molecules. Descriptors are sorted in decreasing order of their SHAP values for [6] helicene.

In order to explore these predictions in detail, Figure 9 presents a tornado plot of the SHAP values for three helicene derivatives that create chiral crystals and are included in our held-out test set. Two TP cases are included: [6]helicene (orange bars) and [7]helicene (brown bars), with predicted probabilities of 0.86 and 0.74, respectively. One FN case is included: [10]helicene (blue bars), with a predicted probability of 0.29. Recall that the predicted probabilities are reported in the range [0,1], where 0 means achiral and 1 means chiral, with a decision threshold of 0.5 (see Table S3 in the SM). As already found above, the SHAP values for the TP cases are mostly positive, while for the FN cases they are mostly negative. The absolute SHAP values for [7] helicene are generally smaller than [6]helicene for most descriptors, in line with the smaller predicted probability. The descriptors that differentiate between the TP and FN samples are those with an opposite sign of their values. The most dominant feature in this respect is fragCpx, mathematically defined by

(1) $$\mathrm{fragCpx}=\left|B^{2}-A^{2}+A\right|+\frac{H}{A},$$

where *A* is the number of atoms, *B* is the number of bonds and *H* is the number of heteroatoms in the studied molecule [33]. This definition implies that by increasing the number of helicene rings, the value of this descriptor increases significantly. Indeed, its value for [6]helicene is 311 while for [10]helicene the value is 879 (hydrogen atoms are excluded in this calculation). A closer look at the trend of fragCpx values in our held-out test set reveals that TN samples with high values of fragCpx display negative SHAP values, pushing CrysChiP towards achiral predictions (see Figure S10 in the SM). While empirically convincing, fragCpx by itself does not explain why the prediction flips from chiral to achiral for [10] helicene. Apparently, the accumulated contribution of fragCpx and the other descriptors with negative SHAP values (Eta_eta, Xc-3d, and BCUTS-1h, see Figure 9), push the model toward achiral classification. Note that ETA_eta and Xc-3d are part of the TN-dominant set of descriptors, and their sign change reflects the change in decision direction of the model. Generally, these descriptors quantify different facets of a molecule’s branching level, charge distribution and connectivity, making them sensitive to structural expansion upon increasing the helicene length.

## 3. Methodology

The design of the AI-driven workflow integrated the following phases: (i) dataset curation and preprocessing, (ii) feature engineering with molecular descriptors, (iii) supervised learning using a neural architecture, and (iv) model evaluation using threshold-independent and threshold-dependent metrics. In what follows, we elaborate on each phase. We note that this workflow outperformed an attempt to apply a stacked meta-learning modeling approach that employed synthetic data generation to mitigate the natural bias found in crystallographic databases by which molecules lacking stereocenters crystallize mainly in non-Sohncke space groups. The Methodology Section S2 in the SM provides additional explanations of our strategy.

### 3.1. The Dataset and Its Cleaning Procedure

Version 5.46 of the CSD [23] was searched for organic crystals of a single type of molecular component of up to 100 atoms. To ensure high-quality data, we included structures with 3D coordinates with a maximal R factor of 0.05. Disordered, powder-based, erroneous, and polymeric structures were excluded. The search resulted in 191,194 crystal structures that were downloaded as a list of CSD refcodes. These were subjected to stereochemical classification using an updated version of the ChiPi code [34,35] which applies the CSD Python API [36] to classify the stereochemistry of molecules in organic crystals according to the Cahn–Ingold–Prelog R/S notation. This classification is used to determine the stereochemical relationships among all molecules within a specified unit cell. As already noted above, while powerful, this approach does not account for cases of axial, planar, or helical chirality, which are not resolved by the CSD Python API. More generally, computational identification of molecules as achiral is not straightforward since some forms of molecular chirality depend on three-dimensional conformation and configurational stability. We therefore used the more concrete and reproducible criterion of the absence of identifiable R/S stereocenters to define the molecular dataset, rather than attempting to classify molecules as achiral in a broader sense. This process identified 106,756 crystals, for which a SMILES string can be defined, as constructed of molecules that lack stereocenters. In total, 11,283 (10.6%) of the crystals crystallize in Sohncke space groups and the rest are found in non-Sohncke space groups. Altogether, this dataset, along with crystallographic properties of the crystals included in the CSD, defined our core dataset used for the model development.

Raw data obtained from crystallographic repositories often contain redundancies, duplicates, and incomplete entries. Therefore, extensive preprocessing of the data was performed before modeling (see SM for more details). The preprocessing workflow followed a structured, multi-phase procedure designed to clean, validate, and standardize crystallographic information. This included the following phases. 1. Defining input and target data columns for the AI model: The SMILES string of the heaviest component of each refcode was used as the input for further analysis. The target columns included a binary 0/1 data designating achiral/chiral crystallization with a non-Sohncke/Sohncke space group, respectively. 2. Conflicting cases in which the same molecule was reported both in Sohncke and non-Sohncke space groups were excluded. 3. Removing duplicate measurements: instances where the same molecule is reported multiple times. For each case of multiple measurements, a single representative entry was selected according to a hierarchical set of criteria prioritizing the most recent publication year, the lowest R-factor, and a study temperature closest to 298 K. Deduplication by both SMILES and Refcode, resulted in a minimal dataset composed only of unique molecular structures. 4. Structural validation: All SMILES strings were checked for validity, and any corrupted or invalid representations were removed. See the Dataset Features Section S2.1 in the SM and Table S8 for more details.

### 3.2. Feature Engineering

RDKit [37] and Mordred [38] were used to compute a comprehensive set of 2D molecular descriptors capturing diverse physicochemical and topological properties. The descriptor space was then cleaned using a three-step pipeline: (1) constant descriptors were removed because they provide no discriminative information and can introduce numerical noise; (2) highly correlated descriptors were pruned to reduce redundancy and multicollinearity and to stabilize downstream learning and interpretation; and (3) all remaining descriptors were standardized to zero mean and unit variance. After this cleaning process, 2563 2D descriptors remained and were used as the candidate feature pool for model development and for subsequent K-best feature selection. We note that the model uses only the above intrinsic molecular descriptors and does not include packing information or any experimental data.

Following preprocessing, the final learning corpus contained 16,000 unique molecules represented by SMILES strings, with a binary target indicating a Sohncke (y = 1) or non-Sohncke (y = 0) space group. The dataset was divided at the molecule level using a stratified split, with no overlap of molecules across the training and held-out test sets. The development set contained 11,200 molecules, balanced as 5600 per class, and the held-out test set contained 4800 molecules, balanced as 2400 per class. Both sets were derived from the same curated CSD source dataset. A single stratified 80:20 split of the development set provided 8960 molecules for weight fitting and 2240 for validation-based early stopping and threshold selection; the supplied implementation does not perform k-fold cross-validation. Throughout this manuscript, “held-out test set” denotes this within-source split; it does not denote an independently collected external validation dataset. The held-out molecules were excluded from fitting the candidate models. However, because the comparisons across K in Table 1 and the associated false negative counts also inform the choice of K = 12 as the representative model, this comparison is descriptive and does not provide an unbiased, independent confirmation of that model selection decision.

Balancing was used to give the two classes equal representation during model fitting and evaluation; it does not reproduce their observed frequency in the CSD source dataset, where 11,283 of 106,756 entries (approximately 10.6%) belong to the Sohncke class. Precision, predictive values, Accuracy, Macro-F1, and PR-AUC can change with class prevalence even when sensitivity and specificity remain unchanged. The no-skill precision baseline for the balanced held-out test set is 0.50, rather than 0.106. ROC-AUC is less directly affected by prevalence under unchanged class-conditional score distributions, but this does not establish transportability to a different chemical population. Accordingly, the values in Table 1 characterize the balanced held-out test set, and the prevalence-adjusted predictive values in Results and Discussion Section 2 are illustrative projections rather than external validation.

Finally, we define K as the number of descriptors retained by K-best feature selection from the cleaned pool of 2563 candidate descriptors. For the top-ranked configurations, descriptors are ranked by mutual information with the label, computed on the 11,200-molecule development set. Because this includes the internal validation subset, that subset is not independent of feature ranking. In the supplied implementation, medians for any remaining missing values are computed before the train/test split; variance and absolute-correlation filtering are fitted on the development set, and scaling is fitted on its 8960-molecule weight-fitting subset. These preprocessing limitations are disclosed rather than assumed to be removed by the present reconciliation of saved predictions.

### 3.3. Supervised Learning Architecture

**Figure 10:**
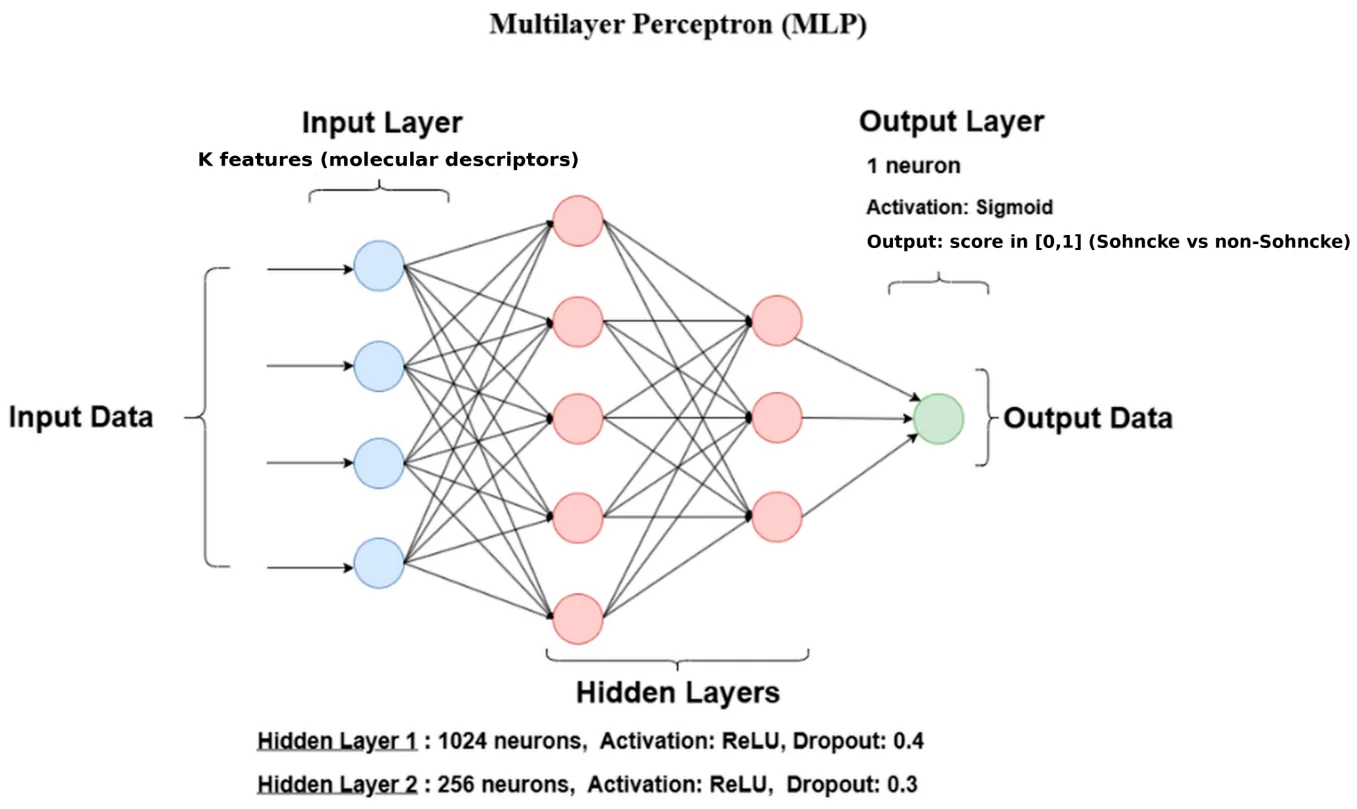
Multilayer Perceptron (MLP) architecture for predicting formation of chiral versus achiral crystals. Schematic not to scale.

Figure 10 illustrates the architecture of the Multilayer Perceptron (MLP) employed in this study to predict whether a molecule with no stereocenters crystallizes in a Sohncke or non-Sohncke space group. We chose this architecture as a practical nonlinear model that can capture interactions between descriptors without becoming overly complex, and that still allows for descriptor-level interpretation through SHAP. The network begins with an input layer, where each molecule is represented by numerical features corresponding to molecular descriptors derived as explained above. The input is then propagated through two hidden layers, each designed to capture nonlinear relationships among descriptors. The first hidden layer comprises 1024 neurons with Rectified Linear Unit (ReLU) activation, and incorporates a drop-out rate of 0.4 to mitigate overfitting. The second hidden layer reduces dimensionality with 256 neurons, also using ReLU activation, and applies a drop-out rate of 0.3 for additional regularization. This hierarchical representation enables the model to learn increasingly abstract patterns that contribute to crystallization outcomes. Finally, the network concludes with a single-neuron output layer activated by the sigmoid function, yielding a probability value $\hat{Y}$ ∈ [0,1]. This output is a Sohncke-class score learned under balanced sampling, with values closer to 1 favoring the positive class and values near 0 favoring the negative class. It should not be interpreted directly as a calibrated crystallization probability at the source prevalence of 10.6% or in another screening population. Together, this architecture combines sufficient modeling capacity with strong regularization, making it well suited to learn the subtle, complex factors that drive chiral or achiral crystallization outcomes.

### 3.4. Model Evaluation

Scoring functions: A variety of scoring functions were employed to assess the performance of the system: Accuracy, Precision, Recall, Macro-F1, ROC-AUC, and PR-AUC. Accuracy measures the proportion of molecules that the model classifies correctly. Precision measures how often predicted positives (chiral, y = 1) are truly positive; Recall measures how many of the true positives are successfully identified; Macro-F1 is the average F1-score across the two classes, giving equal weight to chiral and achiral outcomes; ROC-AUC reflects how well the model separates the classes over the full range of decision thresholds. PR-AUC characterizes the trade-off between Precision and Recall and depends on class prevalence; here, it is evaluated on the balanced held-out test set. Table S1 in the SM specifies the metrics formulas. All metrics ranged from 0 to 1, with a higher value indicating a better score.

Confusion matrix: We used confusion matrices to tabulate the values of TP, TN, FP, and FN for each model. These enable direct inspection of per-class error types, revealing whether misclassifications are primarily due to false positives (overprediction of chiral outcomes) or false negatives (missed chiral outcomes). This decomposition is critical for applications where one type of error (e.g., failing to detect chiral crystals) carries higher scientific or practical consequences.

Decision thresholds: ROC-AUC and PR-AUC are threshold-independent metrics, summarizing how well the model ranks chiral versus achiral crystallization outcomes across all possible cutoffs, whereas Accuracy and Macro-F1 require converting predicted probabilities into class labels using a specified decision threshold. Threshold values were selected on the internal validation subset by maximizing Macro-F1 and then fixed for the confusion matrix-based metrics, and are reported in Table S3 of the SM in order to (i) ensure reproducibility of the reported Accuracy/Macro-F1 values from the raw model scores, (ii) enable fair comparison across K values when calibration differs slightly between models by making the operating point explicit, and (iii) support decision-oriented use, since in practical screening the threshold can be adjusted to balance FN against FP predictions. The separate cost/F1/Youden threshold sweep in Table S4 was optimized on the held-out predictions and is an exploratory sensitivity analysis, not an independently validated operating-point selection process.

Shapley additive explanations: To ensure interpretability of the predictive model, we employed the SHAP [31] methodology, a game-theoretic framework that attributes feature importance by fairly distributing the prediction among input variables. SHAP values *ϕ_j_* quantify the contribution of each feature *j* to the model’s output for a given sample. Formally, they satisfy the additive decomposition

(2) $$f\left(x\right)=\phi_{0}+\sum_{j=1}^{P}\phi_{j}\;.$$

SHAP ensures fairness via the axioms of local accuracy, consistency, and missingness, where *f*(*x*) is the predicted log-odds for input (*x*), *ϕ*_0_ represents the model bias (the expected prediction over the dataset), and *ϕ_j_* corresponds to the marginal contribution of feature *j*. A key advantage of SHAP is that it adheres to the axioms of local accuracy (the sum of feature contributions equals the model prediction), consistency (increasing a feature’s contribution cannot decrease its SHAP value), and missingness (features that are not used by the model receive zero contribution). These properties ensure both fairness and transparency, making SHAP suitable for scientific applications where interpretability is as crucial as predictive performance. In practice, SHAP allows us to move beyond “black-box” neural predictions and identify which molecular descriptors are most influential in distinguishing chiral from achiral crystals. Additional implementation details, computational environment specifications, and runtime profiling are provided in Table S9 in the SM. A complete nomenclature of the variables and abbreviations used in this paper is provided in Table S10.

## 4. Conclusions and Outlook

We provide a solution to the long-standing challenge of predicting when a molecule lacking stereocenters is prone to be crystallized in a chiral crystal structure. We show that this crystallization outcome is an AI-learnable and predictable phenomenon. Starting with organic molecules lacking stereocenters in SMILES notation, CrysChiP—our model—relies on 12 two-dimensional molecular descriptors to predict whether the resulting crystal will belong to a Sohncke or non-Sohncke space group, with accuracy of 0.88 and ROC-AUC of 0.95, far above random baselines (0.5 for ROC-AUC). This result indicates that the descriptor representation captures the link of the two-dimensional molecular structure to its crystallization outcome, even though the underlying process is complex and highly subtle.

We find that, naturally, the relative contribution of individual descriptors varies from one molecule to the next. However, the information they collectively encode points to molecular features associated with chiral crystallization. These include the diversity of atomic environments, the distribution of mass, charge, and ionization potential, as well as topological connectivity and structural complexity (such as branching patterns and the number of structural fragments). Analysis of the held-out test set of 4800 organic molecules from the same curated CSD source, excluded from model fitting, suggests that chiral crystals of molecules lacking stereocenters favor less uniform distributions of these features and greater structural diversity. Moreover, correct predictions of chiral crystals are dominated by a small set of five descriptors (ZMIC3, SM1_Dzm, BCUTZ-1h, bpol, and TIC0), which capture key aspects of mass distribution, electronic topography, bond polarizability, and atom type diversity. Remarkably, while the training dataset did not include molecules with stereocenters, and the model was not given any rules to identify chiral molecules whose chirality arises from other non-superimposable features such as helicity and restricted rotation, it still learned to identify the correct crystallization mode of such structures. Detailed analysis of helicene derivatives demonstrates that ability.

In a recent review, Lin et al. [39] called for more attention to controlled symmetry breaking as a strategy for applications-directed design of molecular crystallization; we believe that our predictive AI model addresses that need. Thus, an important application of our model is the ability to guide the systematic modification of an achiral molecule toward derivatives that eventually provide chiral crystals, offering a practical approach for directing molecular design toward engineered chiral crystals. More generally, our results suggest that AI learning approaches can produce a reliable, reproducible predictive tool for complex properties of molecular structure.

## Figures and Tables

**Table 1:** Model performance as a function of K.

K *	Accuracy	Macro-F1	ROC-AUC	PR-AUC (Chiral)
5	0.515	0.409	0.523	0.515
6	0.854	0.854	0.939	0.941
7	0.859	0.859	0.944	0.947
10	0.864	0.864	0.948	0.949
12	0.876	0.876	0.954	0.956
20	0.885	0.885	0.959	0.963
50	0.890	0.890	0.961	0.964
Max (=2563)	0.887	0.887	0.959	0.961

* The K = 5 configuration is a negative control using low-MI descriptors; K ≥ 6 retains the highest-ranked descriptors. Max uses all candidate features.

**Table 2:** The 12 features of CrysChiP.

Chemical Property	Descriptor Name	Description
Atomic Diversity	TIC0	0-Ordered neighborhood total information content
	ZMIC3	3-Ordered Z-modified information content
Electronic	ATSC0i	Centered Moreau–Broto autocorrelation of lag 0 weighted by ionization potential
	bpol	Bond polarizability
	GGl1	1-ordered raw topological charge
Graph–Spectral (Weighted)	BCUTs-1h	First highest eigenvalue of Burden matrix weighted by intrinsic state
	BCUTZ-1h	First highest eigenvalue of Burden matrix weighted by atomic number
	SM1_Dzm	Spectral moment from Barysz matrix weighted by mass
Structural Complexity	fragCpx	Fragment complexity
	mZagreb1	Modified Zagreb index (version 1)
Topological connectivity	ETA_eta	ETA composite index for reference graph
	Xc-3d	3-Ordered Chi cluster weighted by sigma electrons

**Table 3:** CrysChiP predictions for helicene derivatives.

Prediction	Number of Molecules	% of Molecules
FN	3	12.5%
TN	7	29.2%
TP	14	58.3%
Total	24	100.0%

## Data Availability

The data supporting this article are provided in the Supplementary Materials. In addition, our curated dataset and python codes are available at Zenodo. The processed dataset used for model development and evaluation, including curated labels and molecular descriptor matrices, is available as a CSV file on Zenodo: https://doi.org/10.5281/zenodo.20591103; the CrysChiP python source code is available on Zenodo: https://doi.org/10.5281/zenodo.20590743.

## Supplementary Materials

The following supporting information can be downloaded at https://www.mdpi.com/article/10.3390/molecules31214013/s1: Additional tables and figures of model evaluation and sensitivity data; detailed methodology; nomenclature.

## Abbreviations

The following abbreviations are used in this manuscript:

AI	Artificial Intelligence
AUC	Area Under the Curve
COD	Crystallographic Open Database
CSD	Cambridge Structural Database
CSP	Crystal Structure Prediction
FN	False Negative
FP	False Positive
K-best	Mutual information-based feature selection retaining the K most informative descriptors
Macro-F1	Unweighted average F1 score for chiral and achiral classes
ML	Machine Learning.
MLP	Multilayer Perceptron
Mordred	Python package for high-throughput calculation of molecular descriptors
NPV	Negative predictive value; the fraction of negative predictions that are correct
PDF	Pair Distribution Function
PDFitc	Online AI-based suite for PDF analysis, structure and space-group mining from pair distribution functions
PPV	Positive predictive value; the fraction of positive predictions that are correct
PR	Precision–Recall
PR-AUC	Area under the Precision–Recall curve
RDKit	Open-source cheminformatics library
Refcode	Unique identifier of a crystallographic entry in the structural database (CSD refcode)
ReLU	Rectified Linear Unit
RF	Random Forest
ROC	Receiver Operating Characteristic
ROC-AUC	Area under the ROC curve
SHAP	SHapley Additive exPlanations
SMILES	Simplified Molecular Input Line Entry System
TN	True Negative
TP	True Positive
t-SNE	t-distributed Stochastic Neighbor Embedding
