# Supplementary material for: Predicting Chiral Crystallization of Molecules Without Stereocenters
Source: Molecules. 2026 Nov 6;31(21):4013. doi: 10.3390/molecules31214013 (PMC13764923; doi:10.3390/molecules31214013)
Supplement: Supplementary file 1 [file molecules-31-04013-s001.zip › molecules-4639228-supplementary.pdf]

Supplementary Materials

Predicting Chiral Crystallization of Molecules without Stereocenters

Lior Tobaly[1], Zion Elani[2], Yitzhak Mastai[2], David Avnir[3] and Inbal Tuvi-Arad[4]

[1] School of Business Administration, Bar Ilan University, Ramat-Gan 5290002, Israel

[2] Department of Chemistry and the Institute of Nanotechnology
Bar-Ilan University, Ramat-Gan, 5290002 Israel.

[3] Institute of Chemistry and Center for Nanoscience and Nanotechnology, The Hebrew University of Jerusalem, Jerusalem 9190401, Israel

[4] Department of Natural Sciences, The Open University of Israel, Raanana 4353701, Israel

This Supporting Information provides a comprehensive and reproducible description of the dataset, methodology, and evaluation framework used for predicting chiral crystallization from molecules that lack stereocenters. We detail the construction and curation of a large crystallographic dataset, the computation and preprocessing of high-dimensional molecular descriptors, and mutual information–based feature selection (K-best) for dimensionality reduction. The predictive model is a multilayer perceptron (MLP) trained with focal loss and evaluated under cost-sensitive threshold optimization to account for asymmetric error penalties. Extensive analyses are presented, including confusion matrices across feature subsets, calibration assessment via Brier scores and reliability diagrams, decision-threshold analysis, and stability evaluation across K values. Computational environment specifications and runtime profiling are also provided, demonstrating feasibility for high-throughput and real-time applications. Together, these materials ensure methodological transparency, robustness, and reproducibility of the proposed framework.

Contents

S1. Model Evaluation **3**
S1.1. Model Metrics **3**
S1.2. Confusion Matrices **4**
S1.3. Decision Thresholds **4**
S1.4. Model Sensitivity **5**
S1.4.1. Cost-sensitive analysis and optimal thresholds 5
S1.4.2. Calibration, Brier scores, and probability distributions 8
S1.4.3. Stability across K and error patterns 10
S1.4.4. Confidence, error rates, and selective prediction 12
S2. Detailed Methodology **13**
S2.1. Dataset Features **13**
S2.2. Dataset Preparation **14**
S2.2.1. Molecular Descriptors 15
S2.2.2. Data Preprocessing 15
S2.3. Neural Model Architecture **16**
S2.3.1. Loss Function: Focal Loss 17
S2.3.2. Training Strategy 17
S2.4. Computing Environment **18**
S2.5. Computational Overhead and Real-Time Feasibility **19**
S2.6. fragCpx: SHAP values versus feature values **21**
S3. Additional summarizing and discussion points **21**
S4. The First Modelling Approach **24**
S4.1. Stacked Meta-Learning and why we pivoted to a Neural Network **24**
S4.2. Attempted synthetic data generation and why it did not succeed **24**
S4.3. Why CHIRAL-META underperformed overall **25**
S4.4. Pivot to the Neural Network model **25**
S5. Nomenclature **26**

S1. Model Evaluation

S1.1. Model Metrics

The following metrics were applied in this study, as defined in **Table S1**:

- **Accuracy**: The proportion of correct predictions across all samples.
- **Precision:** The ratio of true positive predictions to all positive predictions.
- **Recall:** The fraction of actual positives correctly identified. Also called True Positive Rate (TPR).
- **F1-Score:** The harmonic mean of the precision and recall scores.
- **Macro-F1**: Average of the F1 scores for the positive and negative classes.
- **ROC-AUC**: The area under the ROC curve, that is the plot of the TPR against the false positive rate (FPR) at varying decision thresholds. A value of 0.5 indicates random guessing, while values approaching 1.0 signify near-perfect discrimination between Sohncke and non-Sohncke classes. ROC-AUC is threshold-independent, making it a robust measure of overall model separability.
- **PR-AUC**: The area under the Precision–Recall (PR) curve, emphasizing the trade-off between precision (the fraction of predicted positives that are correct) and recall (the fraction of actual positives correctly identified). PR-AUC is particularly informative in scenarios with class imbalance, as it highlights the model's ability to capture minority class instances without being dominated by true negatives.

Table S1 : Metrics formulas used for model evaluation.

Metric	Formula
Accuracy	$\frac{TP+TN}{TP+TN+FP+FN}$
Precision	$\frac{TP}{TP+FP}$
Recall (TPR)	$\frac{TP}{TP+FN}$
F1-Score	$\frac{2\cdot TP}{2\cdot TP+FN+FP}$
Macro-F1	$\frac{1}{2}\left(F1_{pos}+F1_{neg}\right)$
FPR (False Positive Rate)	$\frac{FP}{TN+FP}$
ROC-AUC	$\int_0^1 TPR\left(FPR\right)dFPR$
PR-AUC	$\int_0^1 P\left(R\right)dR$

S1.2. Confusion Matrices

Table S2 : Confusion matrices for different values of K.

K	TN (achiral→achiral)	FP (achiral→chiral)	FN (chiral→achiral)	TP (chiral→chiral)
7	2197	203	474	1926
10	2153	247	405	1995
12	2105	295	302	2098
20	2135	265	286	2114
50	2131	269	260	2140
Max	2198	202	340	2060

S1.3. Decision Thresholds

ROC-AUC and PR-AUC are threshold-independent evaluation metrics, meaning that they assess the model's ability to rank chiral crystallization versus achiral crystallization predictions across the full range of possible classification cutoffs. These metrics therefore provide an overall view of discriminative performance without requiring the selection of a single operating point. In contrast, metrics such as Accuracy, Precision, Recall, F1-score, and Macro-F1 require the conversion of predicted probabilities into binary class labels, which can only be done after choosing a specific decision threshold. For this reason, the reported confusion matrices and all threshold-dependent performance measures are directly influenced by the selected cutoff.

The explicit specification of decision thresholds is important for several methodological and practical reasons. First, it ensures reproducibility, since the reported confusion matrices, Accuracy, and Macro-F1 values can be reconstructed exactly from the raw probabilistic outputs of the classifier. Second, it improves transparency and fairness when comparing models trained with different values of K, because even when the same classifier architecture is used, the probability calibration may vary slightly from one feature subset to another. In such cases, reporting the threshold makes the operating point fully visible rather than implicit. Third, threshold selection has practical importance in decision-oriented molecular screening, because the threshold determines the tradeoff between false negatives and false positives. In the present setting, this tradeoff is not merely technical: a lower threshold generally increases sensitivity to chiral molecules, whereas a higher threshold tends to reduce false-positive predictions.

Table S3 summarizes the decision thresholds used to convert predicted probabilities into class labels for the confusion matrices reported in **Table S2**. These thresholds were selected on the validation data by maximizing Macro-F1 and were subsequently held fixed when evaluating the held-out test set. **Table S4** presents a separate threshold-sensitivity analysis in which the operating threshold was independently optimized for expected cost, F1 score, and Youden's J. Consequently, the thresholds reported in **Tables S3** and **S4** are not expected to be identical.

The numerical values in **Table S3** are also informative from an interpretive standpoint. For example, the relatively higher thresholds used for K=6 (0.540) and K=7 (0.550) imply a somewhat more conservative classification rule, meaning that the model requires stronger confidence before assigning a molecule to the chiral class. By contrast, the threshold of 0.480 for K=20

reflects a slightly more permissive operating point, which may be desirable when the objective is to reduce the risk of missing chiral molecules. The threshold for K=12 is exactly 0.500, corresponding to the conventional midpoint decision boundary, whereas the thresholds for K=5, K=50, and the Max model are very close to this value (0.505, 0.505, and 0.515, respectively). Overall, the table shows that although threshold tuning was modest, it was not arbitrary: each value was chosen explicitly to define the operating point used for reporting the final threshold-dependent metrics.

From a broader perspective, **Table S3** reinforces that performance comparison across K values should not rely only on threshold-free measures such as ROC-AUC and PR-AUC but should also consider the threshold at which practical classification decisions are made. Two models may have similar ranking ability yet behave somewhat differently once a fixed decision boundary is applied.

Table S3 : Decision thresholds for different values of K.

K	Features	Fixed threshold
5	5	0.505
6	6	0.540
7	7	0.550
10	10	0.515
12	12	0.500
20	20	0.480
50	50	0.505
Max	2563	0.515

S1.4. Model Sensitivity

S1.4.1. Cost-sensitive analysis and optimal thresholds

In many practical scenarios, false negatives (missing a chiral crystallization) are more costly than false positives. To capture this asymmetry, we set the cost of a false negative to C_FN = 2.0 and the cost of a false positive to C_FP = 1.0, and computed the expected cost as a function of the decision threshold for each value of K. The resulting cost curves for K = 20, 50 and K = Max are shown in **Figure S1** . The reported expected costs describe the balanced evaluation distribution. For a population with positive-class prevalence π, the corresponding population cost is C_FN × π × FNR + C_FP × (1 − π) × FPR; consequently, a cost-optimal operating threshold need not remain optimal when prevalence changes.

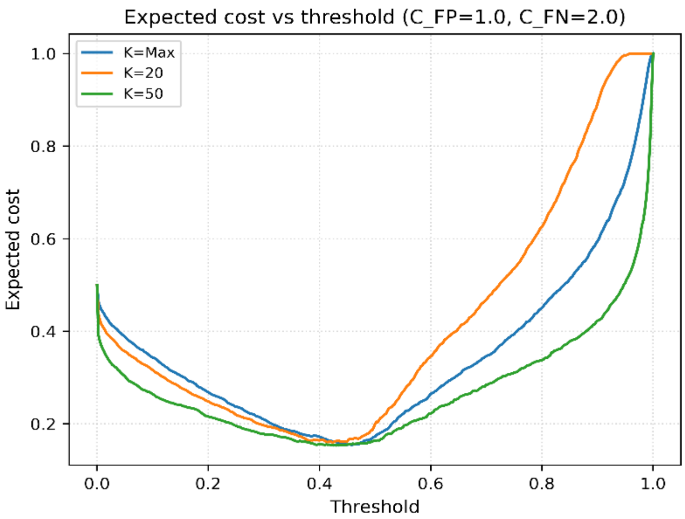

Figure S1 : Expected cost vs threshold (C_FP=1, C_FN=2)

For each value of K, we identified three operating thresholds on a grid of 401 values from 0 to 1: t_cost, which minimizes the expected cost; t_f1, which maximizes Macro-F1; and t_j, which maximizes Youden's J=TPR+TNR−1. The sweep uses the held-out predictions and labels and is descriptive. Its optimized scores should not be interpreted as independent validation of the selected thresholds. The dependence of these thresholds on K is summarized in **Figure S2** and **Figure S3:**

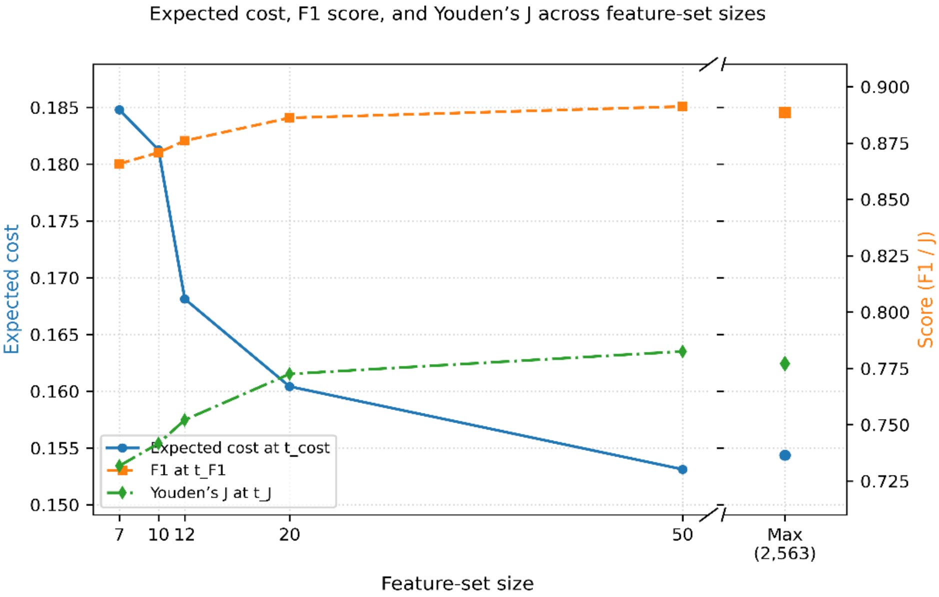

Figure S2 : Expected cost, F1 and Youden's J vs K

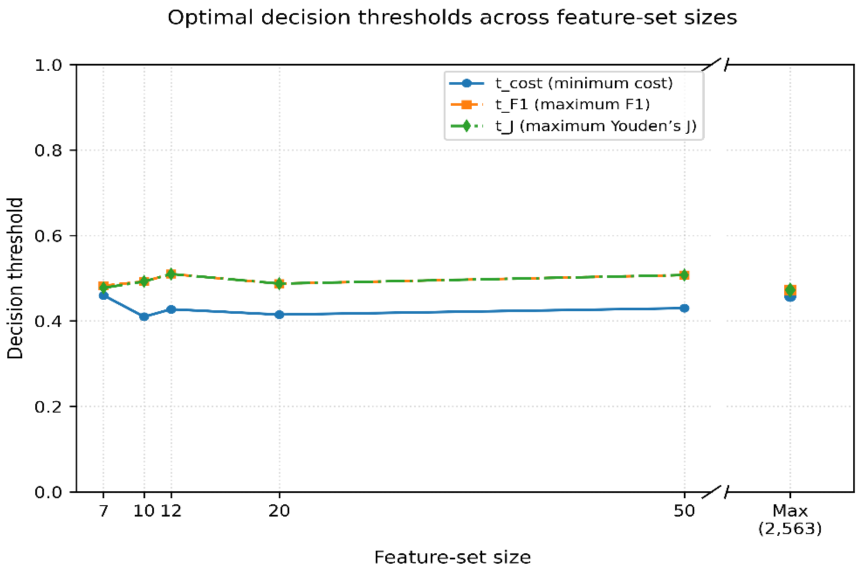

Figure S3 : Optimal thresholds vs K

Table S4 : Cost-optimal thresholds and scores (C_FP = 1, C_FN = 2)

K	t_cost (min cost)	Cost	t_f1 (max F1)	F1 at t_f1	t_j (max J)	J
7	0.4600	0.184792	0.4825	0.865792	0.4775	0.731667
10	0.4100	0.181250	0.4925	0.870830	0.4925	0.741667
12	0.4275	0.168125	0.5100	0.876022	0.5100	0.752083
20	0.4150	0.160417	0.4875	0.886220	0.4875	0.772500
50	0.4300	0.153125	0.5075	0.891250	0.5075	0.782500
Max (=2563)	0.4575	0.154375	0.4725	0.888494	0.4725	0.777083

Table S4 shows the lowest expected cost for K=50 (0.153125), compared with 0.154375 for Max, under C_FP=1 and C_FN=2. K=50 also has the highest optimized Macro-F1 (0.891250) and Youden's J (0.782500) among the tabulated models. Its cost-optimal threshold is 0.4300, compared with 0.4575 for J=2563 (Max). These thresholds were optimized on the held-out predictions for descriptive sensitivity analysis and do not provide an independent estimate of performance after threshold selection.

The DET curves **in Figure S4** provide a complementary view of the trade-off between false-positive and false-negative rates across thresholds. They were regenerated from the same saved prediction scores used for Tables S2–S7; relative model performance depends on the operating region rather than on a uniformly dominant curve.

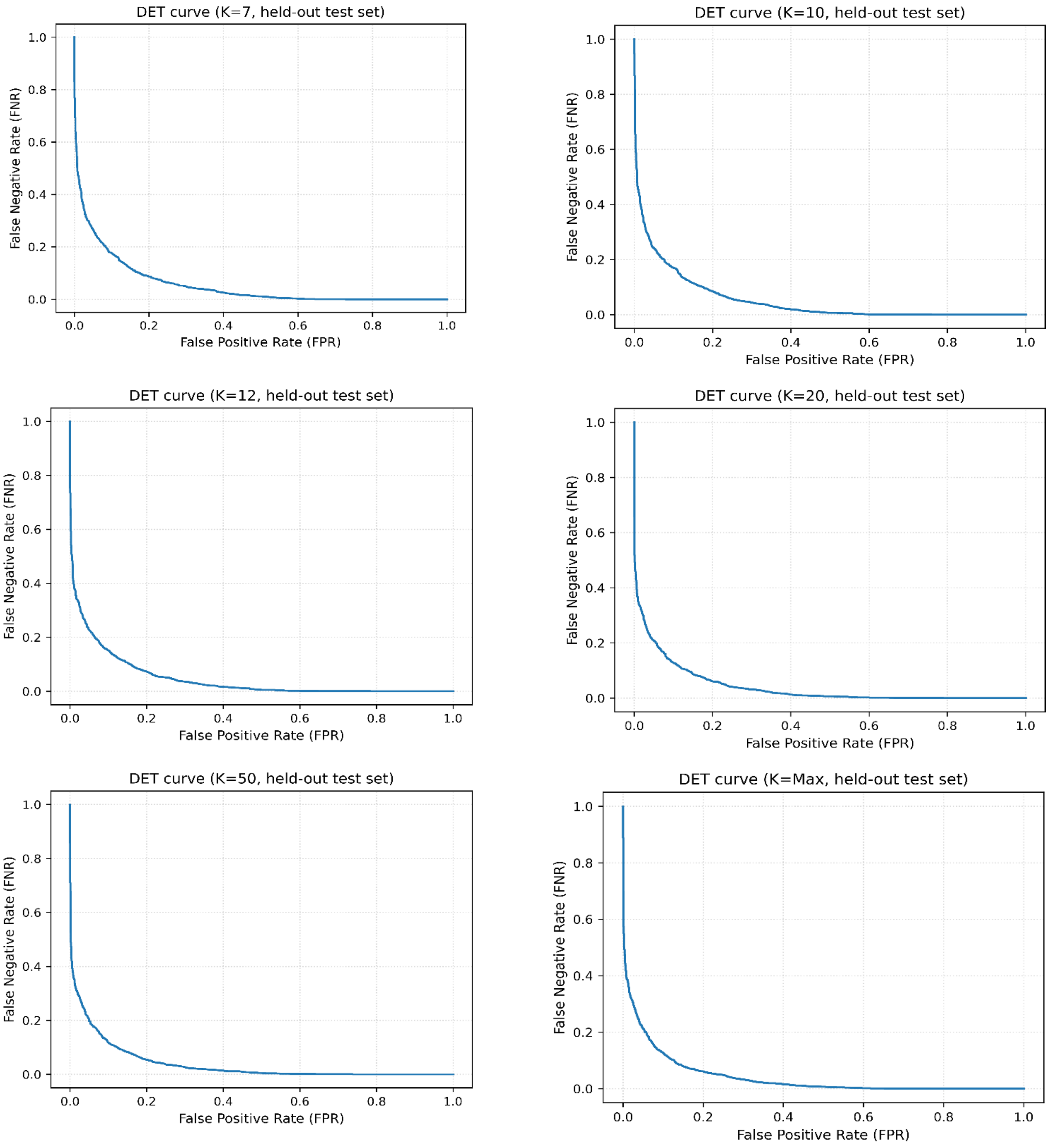

Figure S4 : DET curves of Ks

S1.4.2. Calibration, Brier scores, and probability distributions

Good probabilistic predictions are not only discriminative but also well calibrated. Calibration quality was assessed with Brier scores and reliability diagrams. Brier scores across K are shown in Figure S5 and Table S5. These calibration assessments apply to the balanced held-out test set. Brier scores and reliability diagrams are distribution-dependent and do not establish probability calibration at the 10.6% source prevalence or in an external screening population.

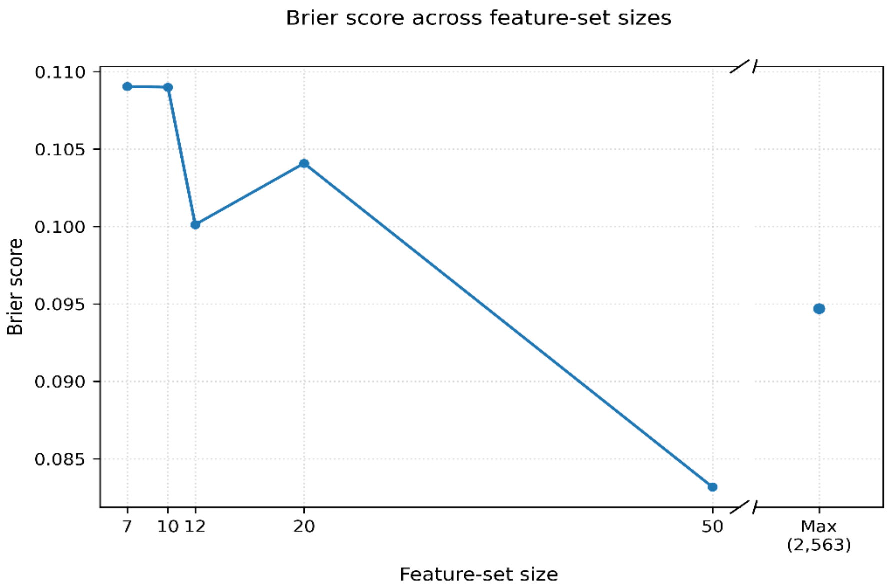

Figure S5 : Brier scores across K (**Figure S5** and presented at **Table S5**)

Table S5: Brier scores by K (held-out test set)

K	Brier score
7	0.109046
10	0.108999
12	0.100123
20	0.104084
50	0.083175
Max (=2563)	0.094705

K=50 has the lowest Brier score among the evaluated models (0.083175), compared with 0.094705 for K=2563 (Max). The scores for K=7, 10, 12 and 20 are 0.109046, 0.108999, 0.100123 and 0.104084, respectively. Brier scores summarize probabilistic prediction error on this balanced evaluation distribution; they do not isolate calibration from discrimination or establish calibration at another prevalence.

Figure S6 compares the empirical reliability curves for K=50 and Max against the ideal diagonal using ten equal-width probability bins. These curves were regenerated from the same saved predictions as Table S5 and show that the observed positive fraction can differ from the mean predicted score within a bin.

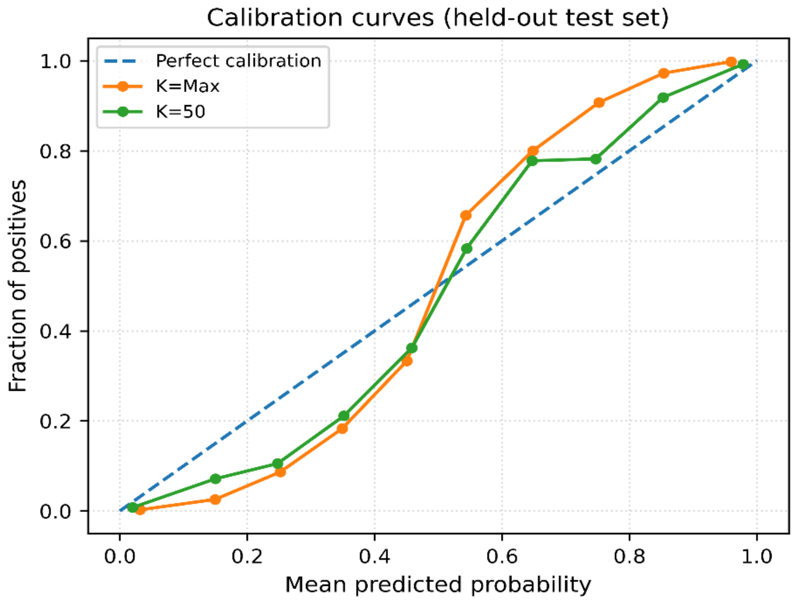

Figure S6: Calibration curves (held-out test set)

S1.4.3. Stability across K and error patterns

Using the saved per-molecule predictions for the six models listed below, we can characterize prediction stability across different K values. Molecules were assigned to one of three categories:

- Always correct: all models (K = 7,10,12, 20, 50, Max) predict the true label correctly.
- Always wrong: all models misclassify the molecule.
- Mixed: predictions vary across K, with at least one correct and one incorrect model.

The stacked bar chart **Figure S7** provides a visual summary, while **Table S6** gives the exact counts.

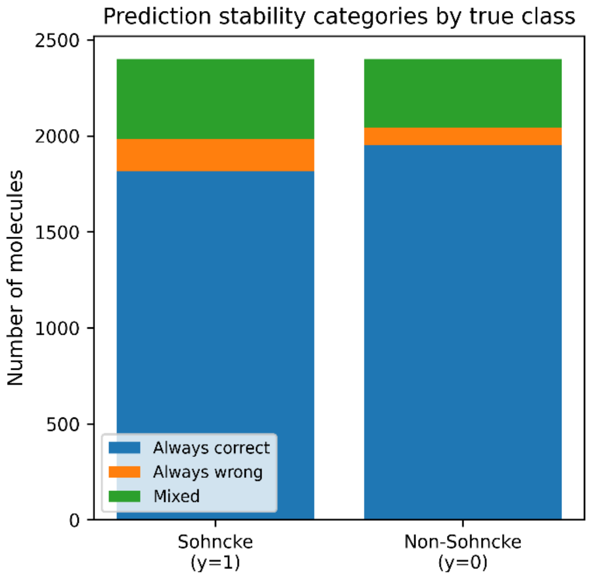

Figure S7: Prediction stability categories by true class

Table S6: Prediction stability across models (held-out test set)

Category	Total molecules	Sohncke (y=1)	Non-Sohncke (y=0)
Always correct	3770	1817	1953
Always wrong	258	167	91
Mixed	772	416	356
Total	4800	2400	2400

Across the six models (K=7, 10, 12, 20, 50 and Max), 3,770 of 4,800 molecules (78.54%) are always classified correctly, 258 (5.38%) are always misclassified and 772 (16.08%) have mixed outcomes. The corresponding Sohncke/non-Sohncke counts are 1,817/1,953, 167/91 and 416/356, respectively. This calculation excludes the low-MI negative controls and uses the fixed per-model reporting thresholds in Table S3.

To compare K-best models with the baseline, we counted, on the same 4,800 held-out molecules, how many of the Max model's 542 errors are corrected and how many of its 4,258 correct classifications become errors. These counts are visualized in **Figure S8** and summarized in **Table S7**. For each K, corrected errors minus new errors equals the difference in correct counts between that model and Max in Table S2.

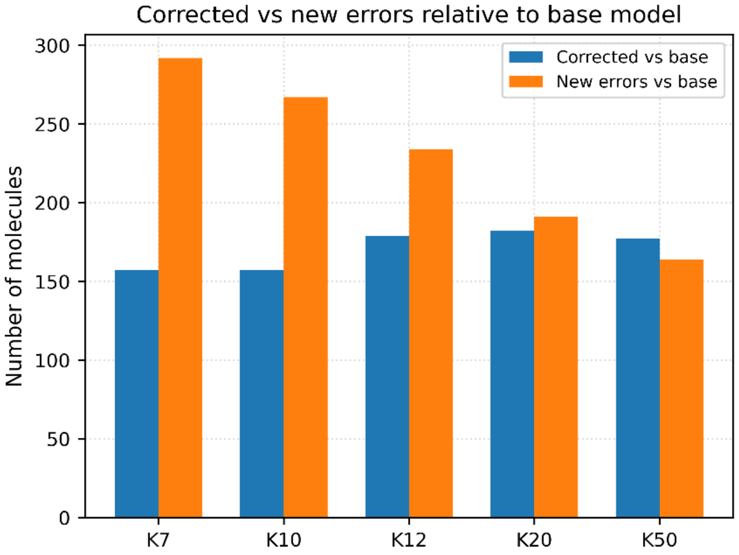

Figure S8: Corrected vs new errors relative to base model

Table S7: Molecules corrected vs newly misclassified relative to the baseline

Model vs base	Corrected base errors	New errors vs base
K7	157	292
K10	157	267
K12	179	234
K20	182	191
K50	177	164

K=7 corrects 157 baseline errors and introduces 292 new errors (net −135); K=10 corrects 157 and introduces 267 (net −110); K=12 corrects 179 and introduces 234 (net −55); K=20 corrects 182 and introduces 191 (net −9); and K=50 corrects 177 and introduces 164 (net +13). Thus, only K=50 has a positive net gain over Max at the fixed reporting thresholds. These paired counts agree exactly with Table S2; they do not establish that K=12 improves overall accuracy over the full-feature baseline.

S1.4.4. Confidence, error rates, and selective prediction

Beyond static thresholding, the model also examines how predicted probabilities are distributed and how well these probabilities align with the observed fraction of positive cases. For each value of K, **Figure S9** presents a paired view consisting of a reliability diagram together with a histogram of prediction frequencies across probability bins. This combination makes it possible to evaluate both the calibration quality of the model and the way its confidence scores are distributed. The observed fractions in these reliability diagrams are those of the balanced held-out test set; the resulting confidence estimates require reassessment before being interpreted as probabilities in a population with a different class distribution.

For each K, the reliability diagram in Figure S9 reports the observed positive fraction against the mean predicted score in ten equal-width bins, and the histogram reports how many predictions fall in each bin. These empirical curves summarize agreement and departures from the ideal diagonal; they do not imply uniformly calibrated probabilities or independent external validation.

Figure S9 uses the same saved score vectors as Tables S2–S7. Its confidence distributions and reliability curves describe the balanced held-out set. Their interpretation as probabilities in a screening population requires reassessing class prevalence and calibration in that population.

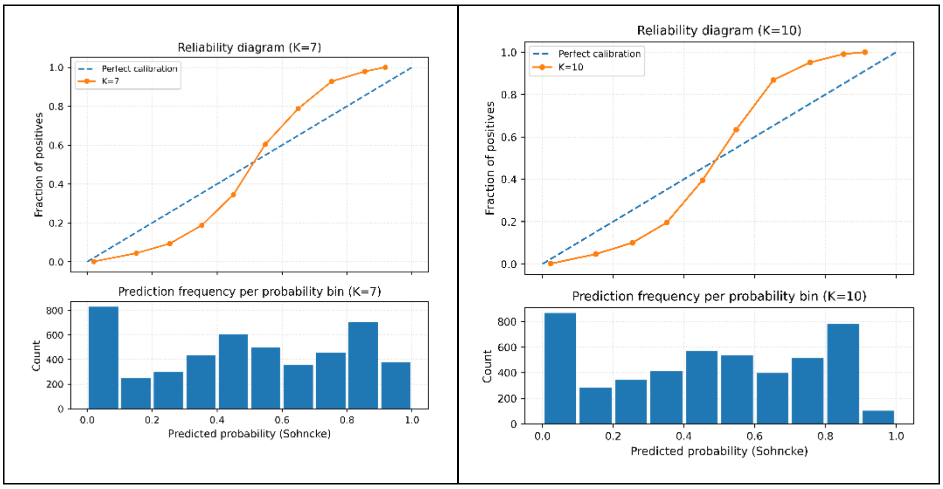

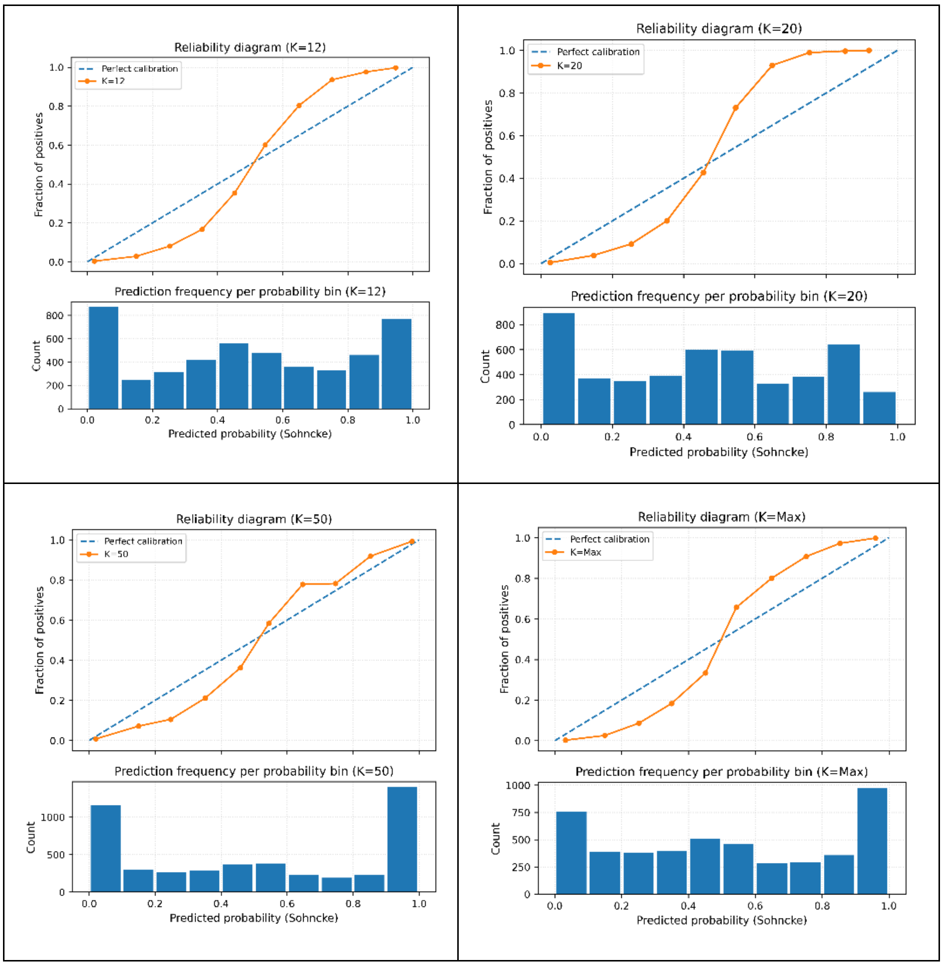

Figure S9 : Prediction Probability and reliability

S2. Detailed Methodology

S2.1. Dataset Features

The dataset used in this study is organized at the level of individual crystallographic entries. Each row corresponds to a single structure, uniquely identified by the Refcode, which allows unambiguous linking back to the original crystallographic database entry without leaking any chemistry-specific information into the model itself.

The target label, “Is Sohncke?”, encodes whether the crystal belongs to a Sohncke space group. In all subsequent modeling, this Boolean field is treated as the ground-truth class:

- True = crystallization in a Sohncke space group
- False = crystallization in a non-Sohncke space group

This framing allows us to directly cast the task as a binary classification problem: “Does this molecule crystallize in a Sohncke space group or not?”

Several additional fields provide experimental and bibliographic context. The R-factor serves as a quality indicator for the refinement of the crystal structure. The original dataset was chosen with R ≤ 0.05. The Study Temperature (in Kelvin) captures the conditions under which the structure was determined. The Publication Year records when the structure was reported and is

used for temporal analyses (e.g., constructing time-aware splits). These factors were later used to clean the dataset and remove duplicates (see the *Data Preprocessing* section below).

For each entry, the SMILES-Heaviest-Component field stores a canonical SMILES string corresponding to the heaviest molecular component in the unit cell of the crystal, extracted using the CCDC python API package. This SMILES string is not fed directly into the neural network; instead, it forms the basis for descriptor computation. Finally, the high-dimensional Molecular Descriptors block aggregates the features used for machine learning. For each SMILES string, we computed a broad panel of molecular descriptors using RDKit and Mordred software, covering constitutional features, atom- and bond-type counts, topological and information-theoretic indices, autocorrelation metrics, ring-system descriptors, and key physicochemical estimators. After descriptor calculation, we applied standard filtering steps (removal of constant or near-constant features, highly correlated descriptors, and those with excessive missing values) followed by scaling to zero mean and unit variance. The resulting descriptor matrix – together with the binary Sohncke label – constitutes the main input to the classification models described in the following sections. The core fields retained for modeling and analysis are summarized in **Table S8**, which lists the crystallographic identifiers, target label, key experimental metadata, and the aggregated block of molecular descriptors. Together, these features define the structured input space used by all subsequent machine-learning experiments in this study.

Table S8: Dataset Features

Feature	Description	Unit
Refcode	CSD Unique identifier	String
Is Sohncke?	Binary label indicating whether the crystal belongs to a Sohncke space group	Boolean (True/False)
R-factor	Measured experimental precision quoted for a crystal	Dimensionless ratios
Study Temp.	Experimental temperature at which the crystal structure was determined	K
SMILES-Heaviest-Component	Canonical SMILES of the heaviest component in the unit cell	String
Publication Year	Year of publication of the crystallographic structure	Year (YYYY)
Molecular Descriptors	Computed 2D molecular descriptors, used as features for ML	Dimensionless (various scales)

S2.2. Dataset Preparation

The construction of a reliable dataset is one of the most critical steps in machine learning applications, especially in domains where experimental data collection is expensive and subject to bias. In this study, the dataset was assembled from the Cambridge Structural Database (CSD), as described in the main manuscript, and contains organic molecules that lack stereocenters. Each molecule was annotated with its observed crystallization outcome: whether it crystallized into a Sohncke space group or a non-Sohncke space group. This binary labelling forms the foundation of our classification task.

Raw data obtained from crystallographic repositories often contain redundancies, duplicates, and incomplete entries. Therefore, extensive data cleaning and deduplication were performed. After this curation process, approximately 95,000 unique molecular entries remained. To ensure that the classifier would not become biased toward the more frequent class, we constructed balanced subsets. Specifically, 8,000 chiral crystals and 8,000 achiral crystals were randomly selected for training and evaluation. This balanced design reduces the skew in class distribution and facilitates the training of models that are sensitive to minority class detection. The resulting 16,000-molecule learning corpus was split at the molecule level into 11,200 development molecules (5,600 per class) and 4,800 held-out test molecules (2,400 per class), with no overlap of molecules. Both sets were drawn from the same curated source. The approximately 10.6% Sohncke-class prevalence quoted in the main manuscript refers to the 106,756-entry retrieved source dataset before subsequent curation and balanced sampling; it is not the class prevalence of the balanced learning corpus and need not represent every screening population. Precision, predictive values, Accuracy, Macro-F1, and PR-AUC can change with prevalence even if sensitivity and specificity remain unchanged.

Formally, the dataset can be expressed as:

$$\mathcal{D} = \{(\mathcal{X}_i, \mathcal{Y}_i)\}_{i=1}^{N}, \qquad \mathcal{X}_i \in \mathbb{R}^{\mathcal{P}}\,,\ \mathcal{Y}_i \in \{0,1\} \tag{1}$$

where N=16,000, $\mathcal{P}$ is the feature dimension, and $\mathcal{Y}_i$=1 denotes crystallization in a Sohncke space group while $\mathcal{Y}_i$=0 denotes an achiral crystallization outcome.

S2.2.1. Molecular Descriptors

Feature representation lies at the core of machine learning performance. Molecules are inherently graph-like objects, composed of atoms (nodes) and bonds (edges). To feed them into a classifier, they must be mapped into vectorized descriptors that capture relevant structural and physicochemical properties. Each molecule m_i in the dataset is represented as a structured vector ($\mathcal{X}_i$) of numerical descriptors that capture its chemical and structural characteristics. Formally, this can be expressed as:

$$\mathcal{X}_i = [d_1(m_i), d_2(m_i), \dots , d_k(m_i)] \tag{2}$$

where d_j is the jth descriptor for molecule m_i.

S2.2.2. Data Preprocessing

Raw molecular descriptors required a rigorous preprocessing pipeline to guarantee both numerical stability and meaningful input to the machine learning framework. Without careful preprocessing, the high-dimensional descriptor space could introduce noise, redundancy, or scaling issues that degrade model performance. To address this, several sequential steps were applied.

Variance Filtering: Descriptors exhibiting zero variance across the development-set molecules were eliminated, as they provide no discriminatory information. Formally, a descriptor d_j was discarded if:

$$\mathrm{VAR}(d_j) = 0 \ \Rightarrow \ d_j \text{ dropped.} \tag{3}$$

This ensures that only features capable of differentiating between samples remain in the dataset.

Correlation Pruning: To reduce redundancy and multicollinearity, pairs of highly correlated features were examined. Descriptors with an absolute Pearson correlation coefficient above a threshold (|r|>0.95) were considered redundant, and one from each correlated pair was removed:

$$\rho_{jk} = \frac{\mathrm{Cov}(d_j, d_k)}{\sigma_j \sigma_k} \quad (4)$$

where σ_j and σ_k are the standard deviations of descriptors d_j and d_k. This step ensures that the retained feature set is compact yet informative.

Imputation of Missing Values: Computation of certain descriptors occasionally failed due to edge cases in molecular structure. In the supplied implementation, any remaining missing values were imputed using feature medians computed before the train/test split:

$$d_j^{imp} = median(\{d_j\}_{i=1}^{N}) \quad (5)$$

The median was chosen over the mean to mitigate the influence of outliers. Computing it before splitting can allow information from held-out covariates into preprocessing; the effect cannot be assessed from saved predictions without the descriptor matrix and missingness records.

Scaling and Normalization: Since neural networks are sensitive to the magnitude of input features, the scaler was fitted on the 8,960-molecule weight-fitting subset and applied unchanged to validation and held-out test data:

$$\tilde{d}_{ij} = \frac{d_{ij} - \mu_j}{\sigma_j} \quad (6)$$

where μ_j and σ_j represent the mean and standard deviation of descriptor d_j, respectively. This ensures that all features contribute equally during gradient-based optimization.

Variance and absolute-correlation filters were fitted on the 11,200-molecule development set. Mutual-information ranking was also fitted on this set, including its 2,240-molecule internal validation subset, so that subset is not independent of feature selection. In the preprocessing expressions, the variance/correlation indices refer to the development set, scaling moments to the weight-fitting subset, and imputation medians to the input descriptor table before splitting. The present reconciliation recomputes summaries from saved predictions; it does not refit preprocessing or retrain models.

S2.3. Neural Model Architecture

We employed a Multilayer Perceptron (MLP) Neural Network with two hidden layers, Rectified Linear Unit (ReLU) activation, and dropout. This choice reflects the non-linear nature of stereochemical effects while providing computational efficiency. Forward propagation is defined as:

$$\boldsymbol{h}_1 = \mathrm{ReLU}(W_1 x + \boldsymbol{b}_1), \ \boldsymbol{h}_1 \in \mathbb{R}^{1024} \quad (7)$$

$$\boldsymbol{h}_2 = \mathrm{ReLU}(W_2 \boldsymbol{h}_1 + \boldsymbol{b}_2), \ \boldsymbol{h}_2 \in \mathbb{R}^{256} \quad (8)$$

$$z = W_3 \boldsymbol{h}_2 + \boldsymbol{b}_3 \quad (9)$$

$$\hat{y} = \sigma(z) \quad (10)$$

where $\sigma(z) = \frac{1}{1+e^{-z}}$ is the logistic sigmoid mapping to probabilities. Dropout layers with rates $\mathcal{P} = 0.4$ and $\mathcal{P} = 0.3$ were inserted after each hidden layer to mitigate overfitting.

S2.3.1. Loss Function: Focal Loss

Although the dataset was deliberately balanced between the two classes, it still contained hard-to-classify molecules. Focal Loss was therefore used to reduce the contribution of confidently classified examples and place greater emphasis on hard or uncertain cases. Unlike conventional Binary Cross-Entropy (BCE), Focal Loss applies a confidence-dependent modulating factor that further down-weights well-classified examples. The standard BCE loss is defined as:

$$\mathcal{L}_{BCE} = -(\mathcal{Y}\, log\mathcal{P} + (1-\mathcal{Y})\log(1-\mathcal{P})) \quad (11)$$

where $\mathcal{Y} \in \{0,1\}$ is the ground truth label and $\mathcal{P}$ is the predicted probability of the positive class. While BCE is widely used in binary classification, it tends to be dominated by easily classified examples, which can hinder learning in imbalanced datasets where one class is underrepresented. To address this, Focal Loss introduces a modulating factor $(1-\mathcal{P}_t)^{\gamma}$ that down-weights well-classified instances and forces the model to focus more on misclassified or uncertain cases:

$$\mathcal{L}_{\text{focal}} = -\alpha(1-\mathcal{P}_t)^{\gamma}\log(\mathcal{P}_t) \quad (12)$$

where:

$$\mathcal{P}_t = \begin{cases} \mathcal{P}, & \text{if } \mathcal{Y} = 1 \\ 1-\mathcal{P}, & \text{if } \mathcal{Y} = 0 \end{cases} \quad (13)$$

Here α is a balancing parameter that adjusts the relative importance of positive and negative classes, and γ is a focusing parameter that controls how strongly easy examples are down-weighted. In this study, α = 0.5 was used to assign equal weight to the two classes, consistent with the balanced dataset, while γ = 2.0 was selected to down-weight confidently classified examples, and focus learning on harder or more uncertain cases.

S2.3.2. Training Strategy

To ensure reliable and generalizable performance, a carefully designed training strategy was employed, including the following steps.

Internal validation split: The 11,200-molecule development set was split once using a stratified 80:20 partition, giving 8,960 molecules for fitting network weights and 2,240 for validation-based early stopping and threshold selection. The supplied evaluation code uses one such split and does not implement 5-fold cross-validation. The 4,800-molecule held-out test set was excluded from weight fitting, while its results informed the descriptive comparisons across K.

Optimizer: Training was conducted using the AdamW optimizer, a variant of Adam that decouples weight decay from the gradient update process. This choice was motivated by its

strong empirical performance in deep learning tasks and its ability to reduce overfitting through regularization. The optimizer employed a learning rate of $\eta=3\times10^{-3}$, which provided a balance between fast convergence and stable updates. A weight decay coefficient of 10^{-4} was applied to penalize large parameter magnitudes, further improving generalization by discouraging overly complex solutions.

Early Stopping: To prevent overfitting and unnecessary computation, an early stopping mechanism was integrated. Training was terminated if the validation ROC-AUC failed to improve over 20 consecutive epochs. This criterion was chosen because ROC-AUC directly reflects the model's discriminative power in distinguishing chiral from achiral crystallization outcome, making it a more informative measure than accuracy in imbalanced settings. Early stopping ensures that the final model captures meaningful descriptor–label relationships without memorizing noise from the training set.

Batching and Regularization: Training was performed in mini-batches (size 256), enabling efficient GPU utilization and smoothing gradient updates. In addition, dropout layers (with probabilities 0.4 and 0.3 across hidden layers) acted as a stochastic regularizer, reducing co-adaptation of neurons and improving robustness.

Performance Tracking: For each model configuration, metrics such as accuracy, F1-score, ROC-AUC, and PR-AUC were recorded to evaluate both class-specific and overall performance. The reporting thresholds in Table S3 were optimized on validation data after training and then held fixed for evaluation of the held-out test set. The separate cost and threshold-sensitivity analyses characterize the specified evaluation distribution; they do not establish an optimal threshold for a new screening population.

Threshold optimization: The fixed $\hat{y} \in [0,1]$ reporting thresholds in Table S3 were selected by maximizing validation Macro-F1. The separate sensitivity analysis in Table S4 searches the held-out scores for thresholds minimizing misclassification cost:

$$\mathrm{Cost(t)} = \frac{\mathrm{C_{FP}} \cdot \mathrm{FP}(t) + \mathrm{C_{FN}} \cdot \mathrm{FN}(t)}{N} \quad (14)$$

with $\mathrm{C_{FP}} = 1.0$, $\mathrm{C_{FN}} = 2.0$ emphasizing the penalty of false negatives. The held-out sweep also computes thresholds maximizing Macro-F1 and Youden's J-statistic. These post-hoc optimized scores are descriptive and are distinct from the fixed-threshold results in Table S2.

S2.4. Computing Environment

All experiments were conducted on a dedicated workstation with the following specifications:

Hardware:

- CPU: Intel Core i9 (12 cores @ 3.7 GHz)
- RAM: 64 GB DDR4
- GPU: NVIDIA RTX 3080 Ti with 12 GB VRAM (used for MLP training and SHAP acceleration)

- Storage: 2 TB NVMe SSD

Software:

- Operating System: Windows 11 (Version 24H2 OS Build 26100.6584)
- Python 3.13
- Key Libraries:
    - **PyTorch 2.2.0** (MLP training, GPU acceleration)
    - **scikit-learn 1.5.1** (feature selection, metrics, preprocessing)
    - **RDKit 2024.03** (molecular descriptor calculation)
    - **Mordred 1.2.0** (molecular descriptor calculation)
    - **SHAP 0.44.1** (explainable AI analysis)
    - **NumPy / Pandas / Matplotlib** (data handling and visualization)

To ensure reproducibility, all experiments were executed in a controlled Python environment with pinned library versions, and random seeds were fixed for NumPy, PyTorch, and scikit-learn wherever applicable. The same preprocessing and model-training pipeline was applied across the model configurations and experimental variants, so that performance differences can be attributed to changes in model architecture, feature sets, or decision thresholds rather than to implementation artifacts. Data loading, descriptor computation (RDKit/Mordred), and model evaluation were orchestrated via a single script-driven workflow to minimize manual intervention and reduce the risk of configuration drift.

The GPU (RTX 3080 Ti) was used for training the multilayer perceptron models and for accelerating SHAP value computation, which substantially reduced the runtime of both hyperparameter exploration and explainability analyses compared to CPU-only execution. Descriptor calculation and classical machine-learning steps (feature selection, calibration, metrics) were carried out on the CPU, leveraging vectorized NumPy/pandas operations to handle the high-dimensional descriptor matrices efficiently.

S2.5. Computational Overhead and Real-Time Feasibility

To evaluate the practicality of our framework, we measured the runtime of each major pipeline component on the described workstation.

Table S9: Runtime Breakdown for the System

Component	Runtime (ms), reported estimate
Data preprocessing (scaling, filtering)	~35 ms
RDKit/Mordred molecular descriptor computation	~120 ms
MLP training (early-stopped)	~2,800 ms
Feature selection (K-Best, MI)	~40 ms
SHAP explainability (top 50 features)	~3,200 ms

These results emphasize several important observations. The preprocessing stage, which includes scaling, variance-based filtering, and removal of highly correlated features, requires

less than 50 ms to complete and therefore has a negligible impact on real-time performance. The computation of molecular features, specifically the calculation of RDKit and Mordred molecular descriptors, introduces an additional delay of approximately 120 ms. No chirality-specific filtering was applied at this stage; the full descriptor set was calculated independently of any presumed relationship to chirality, and the most informative descriptors were subsequently identified using mutual-information-based feature selection. This computation is performed once for each data batch, and its runtime scales with the number of molecules processed.

Model training is also efficient. The multilayer perceptron (MLP) reaches convergence in about 2.8 s in the previously reported timing estimate, which means that retraining remains practical even on relatively modest computing hardware. As a result, the system can be updated frequently as new crystallographic information becomes available. The cost of feature selection, carried out using a mutual-information-based "K-Best" procedure, adds only around 40 ms, indicating that this form of dimensionality reduction can be applied without introducing meaningful runtime overhead.

The most computationally demanding component is the explainability analysis. The SHapley Additive exPlanations (SHAP) method requires approximately 3.2 s to complete. Since this analysis is performed offline and is intended mainly for interpretability and the identification of informative features, it does not constrain real-time system performance. In practical terms, the entire production-level inference pipeline, which includes both preprocessing and model prediction, finishes well within 200 ms for each batch, demonstrating that the system fully supports real-time operation in high-throughput screening environments.

S2.6. fragCpx: SHAP values versus feature values

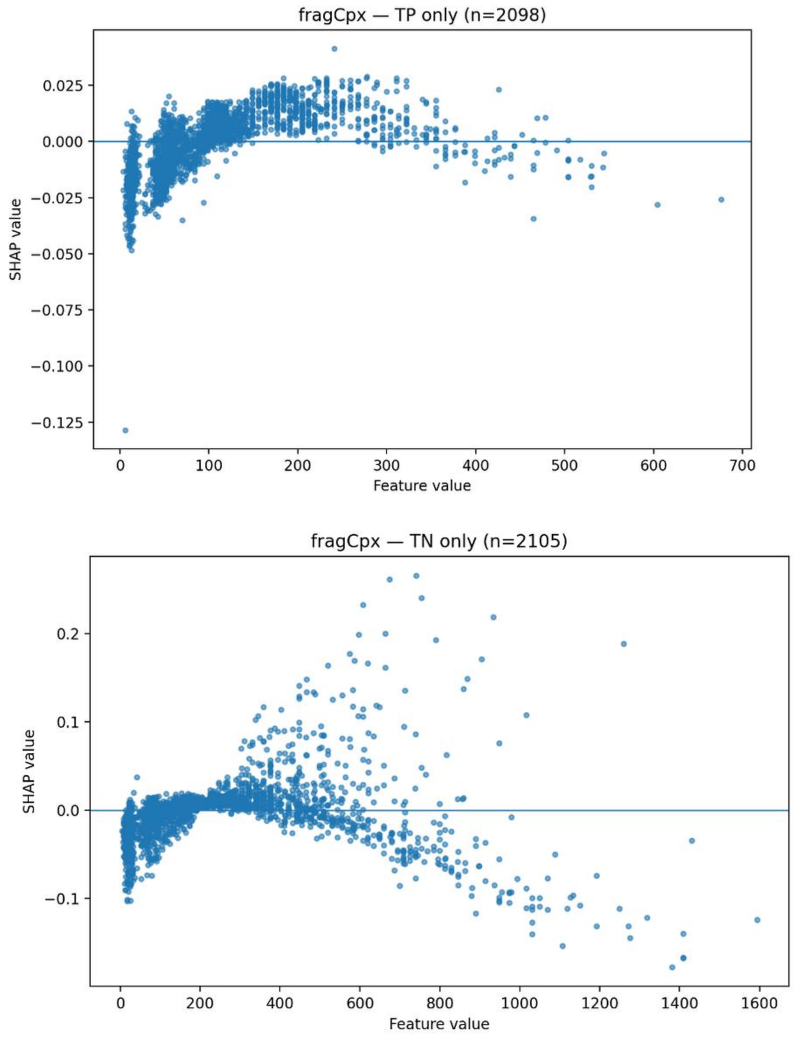

Figure S10 : SHAP values versus feature values for fragCpx for the held-out test set: Top: TP samples. Bottom: TN samples.

S3. Additional summarizing and discussion points

From an AI perspective, this study demonstrates that the crystallization outcome, whether a molecule lacking stereocenters crystallizes into a chiral or an achiral form, is not only learnable from molecular descriptors, but learnable in a way that is consistent, quantitatively strong, and realistic to operationalize in chemistry-facing workflows. The central message in the current narrative is therefore not an exhaustive exploration of how performance varies across different K values, but rather the justification and characterization of a single representative model that offers a strong balance between predictive power, compactness, and reproducibility. In that spirit, we focus on K=12, i.e., a model trained on a compact set of 12 MI-selected descriptors, as the representative configuration used to communicate the method's practical capability.

The performance of the K=12 model on a balanced held-out test set from the same curated CSD source indicates that the descriptor-based pipeline captures a strong and stable signal linking molecular structure to crystallization outcome. Quantitatively, the held-out test-set ROC-AUC is 0.9538, and the corresponding PR-AUC for the chiral class is 0.9564 (with PR-AUC of 0.9547 for the achiral class). These AUC values are far above the ≈0.5 baselines expected under random guessing and, in practical terms, mean that the model ranks true chiral crystallization ahead of true achiral crystallization outcomes with high reliability across a wide range of thresholds. In addition to threshold-free ranking metrics, the K=12 model also yields strong point performance at a conventional operating threshold: using a 0.5 decision threshold, the held-out test-set accuracy is 0.8756 and the macro-F1 is 0.8756. For chemists, this matters because it indicates that the model does not achieve its AUC by "sacrificing" one class; rather, it maintains balanced performance across chiral and achiral crystallization outcomes when forced to commit to a hard decision.

The confusion matrix at this operating point provides an intuitive, concrete view of how errors are distributed in the held-out test set. With rows corresponding to true classes and columns to predicted classes (achiral/chiral), the K=12 test confusion matrix is [[2105, 295], [302, 2098]]. In other words, among the 2400 molecules that form achiral crystals in the held-out test set, 2105 are correctly predicted to form achiral crystals while 295 are predicted to form chiral crystals; among the 2400 structures with Sohncke-class labels, 2098 are correctly predicted to form chiral crystals while 302 are predicted to form achiral crystals. This near symmetry is exactly what one hopes to see in a balanced evaluation: the model is not "biased" toward one output category in a way that would artificially inflate performance. Consistent with this, the class-specific precision/recall values for the chiral class are also balanced and strong, with precision = 0.8767, recall = 0.8742, and F1 = 0.8754 at the 0.5 threshold. In the balanced held-out test set, about 88% of positive predictions are correct and about 87% of positive cases are identified. The first percentage depends on class prevalence and should not be interpreted as the expected screening precision at 10.6% prevalence. Assuming unchanged sensitivity (2,098/2,400) and specificity (2,105/2,400), prevalence-adjusted positive predictive value (PPV) = $Se \times \pi / [Se \times \pi + (1 - Sp) \times (1 - \pi)]$ and NPV = $Sp \times (1 - \pi) / [(1 - Se) \times \pi + Sp \times (1 - \pi)]$ give approximately 45.7% and 98.3%, respectively, at $\pi = 0.106$. These are illustrative projections, not results from an external or naturally distributed evaluation cohort.

Equally important as the raw metrics is what they imply about generalization. In ML terms, high test-set performance is only valuable if it reflects a genuine structure–outcome relationship rather than a shortcut caused by data leakage, near-duplicates, or overly permissive splitting. Here, the evaluation is explicitly performed on a held-out test set from the same curated CSD source with explicit separation between train and test molecules, together with the curation protocol and a single internal validation split during model development. Molecule-level separation prevents identical molecules from occurring in both sets, but does not by itself rule out closely related analogs across the split or establish generalization to novel scaffolds or an independently collected dataset. The K=12 results support a structure–outcome association within this source population. Because the reported comparisons across K and error counts also inform selection of the representative model, the comparison is descriptive rather than an unbiased, independent confirmation of that selection decision. Independent external validation remains future work.

The choice to center the narrative on K=12 is also motivated by practical considerations. For many chemists, a model that depends on thousands of descriptors can be difficult to operationalize, interpret, and maintain across toolchains, databases, and collaborators. A compact descriptor set is easier to reproduce, easier to audit, and less likely to be sensitive to subtle differences in descriptor implementation, missing values, or version drift in descriptor calculators. The K=12 configuration therefore represents a pragmatic and scientifically defensible compromise: it is small enough to be manageable, but still sufficiently expressive to preserve high discriminative power, as reflected by ROC-AUC ≈ 0.954 and PR-AUC ≈ 0.956. In this paper's framing, K=12 is thus treated as the recommended "representative operating point" rather than merely one point on a performance curve.

From the standpoint of deployment-readiness, another key aspect of the K=12 results is the coexistence of strong ranking performance (AUCs) with strong fixed-threshold performance (accuracy/macro-F1). AUCs tell us that the model's ordering of molecules by predicted propensity is reliable; accuracy and macro-F1 tell us that when a binary choice must be made at a specified threshold, the model remains effective and balanced. In real experimental settings, both properties matter. Researchers might use the model in a ranking mode to prioritize a short list of candidates for expensive crystallization trials, or they might use it in a binary mode to flag molecules that appear strongly likely to crystallize in one mode versus the other. The K=12 results motivate both usage modes within the evaluated population. Practical screening requires checking class prevalence, calibration, the operating threshold, and performance in the intended population.

It is also useful to interpret these outcomes in relation to the fundamental "random baseline." In balanced binary classification, random guessing yields ROC-AUC ≈ 0.5 and PR-AUC close to the class prevalence. In the balanced held-out test set, that PR baseline is also near 0.5. The observed K=12 ROC-AUC of 0.9538 and PR-AUC of 0.9564 therefore represent not a marginal improvement but a large separation from chance, implying that the descriptor representation contains substantial predictive information about crystallization chirality. This is a meaningful scientific claim: it supports the hypothesis that there are consistent structural correlates, encoded in molecular descriptors, that accompany chiral versus achiral crystallization outcomes, and that supervised learning can extract and exploit these correlates in a reproducible way.

Finally, this K=12-centered framing aligns naturally with a forward-looking, application-driven roadmap. A compact, stable model is precisely the kind of artifact that can be packaged for broader access, including via a future online interface where users can submit structures and receive predictions in a standardized way. In such a setting, reproducibility and robustness are not abstract virtues; they are necessities. A versioned K=12 pipeline (fixed descriptor definitions, fixed preprocessing, fixed trained weights) can be deployed and maintained with clear traceability, enabling different labs and collaborators to obtain consistent predictions from the same input structures. The results reported here provide a quantitative justification for that direction: K=12 achieves ROC/PR AUC ≈ 0.95–0.96 and accuracy/macro-F1 ≈ 0.876 on the balanced held-out test set, motivating prospective assessment of its utility in real screening scenarios.

In summary, the K=12 results support the main AI claim of the paper in a chemist-relevant way: chiral crystallization outcome can be predicted from molecular descriptors with high discriminative power and balanced classification performance on a balanced held-out test set

from the same curated CSD source. The reported metrics, accuracy 0.8756, macro-F1 0.8756, ROC-AUC 0.9538, PR-AUC 0.9564 (chiral), and the balanced confusion matrix [[2105, 295], [302, 2098]], collectively indicate a strong, reproducible structure–outcome signal and a model configuration that is realistic to communicate, reproduce, and ultimately deploy as a decision-support component in practical crystallization workflows. These results characterize within-source discrimination under balanced sampling; they do not establish independent external validation or prevalence-specific screening precision.

S4. The First Modelling Approach

S4.1. Stacked Meta-Learning and why we pivoted to a Neural Network

Our first full solution was the CHIRAL-META pipeline: a stacked meta-learning framework built for descriptor-based prediction of molecular crystallization behavior. The problem was formulated as a supervised classification task over a high-dimensional tabular representation, where each molecule is encoded by a large set of computed molecular descriptors. Rather than relying on a single estimator, CHIRAL-META was designed as a modular system whose components could be tuned independently: a feature selection stage to control dimensionality, a pool of diverse base learners to capture complementary decision patterns, and a final meta-learner trained on the base learners' predicted probabilities. At the architecture level, the pipeline was defined as: (1) feature ranking by mutual information, (2) training ensemble base models, (3) meta-model stacking, (4) iterative refinement, and (5) ELI5-driven explainability.

The KBest stage was used as a statistical feature-selection step. Descriptor relevance was quantified using mutual information with the target class, and the top K descriptors were retained using K-best ranking. Following feature selection, we trained a broad set of base models to intentionally introduce model diversity-tree-based baselines, distance-based methods, gradient-boosting families, bagging ensembles, interpretable rule learners, and anomaly-detection style models are all enumerated as part of the base-model suite. The key CHIRAL-META idea was then to convert model diversity into features: each molecule's representation at the meta level is the vector of base-model probability outputs, which are merged into a "meta dataset," and a meta learner is trained on that dataset to produce the final prediction.

In addition to stacking, CHIRAL-META incorporated explicit "quality control" logic intended to reduce noise in training. An iterative refinement mechanism was applied that only reuses samples with confident predicted probabilities - keeping cases where the predicted probability is high for one label or very low for the other label - to strengthen the training signal and suppress ambiguous cases.

S4.2. Attempted synthetic data generation and why it did not succeed

A major part of the early CHIRAL-META effort was the attempt to improve data coverage via synthetic sample generation. We experimented with generating additional labelled samples using CTGAN-like tabular synthesis and then applying distance-based validation to retain only synthetic points that remained close to the real-data manifold. Overall, this strategy generated a large number of synthetic samples.

In practice, this outcome reflected the core limitation of descriptor-space synthesis: producing points that are statistically plausible is not the same as producing points that are chemically meaningful or that preserve the true label-generating mechanisms. Even after filtering, synthetic descriptors can (a) violate implicit chemical constraints that are not explicitly encoded in the tabular features, (b) distort higher-order correlations among descriptors that matter for generalization, and (c) introduce subtle label noise because the generation process does not "know" the causal physical/chemical pathway that connects structure, crystallization behavior, and the descriptors. The nearest-neighbor filter mitigates this only partially: if the threshold is strict, most generated points are discarded (as we observed), and if it is relaxed, more synthetic points pass but the risk of unrealistic or distribution-shifting samples increases. This trade-off meant that synthetic augmentation did not deliver reliable, repeatable improvements in the evaluation settings we cared about, and it sometimes amplified pipeline sensitivity rather than reducing it.

S4.3. Why CHIRAL-META underperformed overall

Although CHIRAL-META is a reasonable and well-motivated design, our experiments led us to treat it as insufficiently reliable for the project's next phase. The primary issue was that the stacked pipeline had too many interacting degrees of freedom: the selected descriptor subset (KBest), the exact composition and tuning of the base-model set, the synthetic augmentation policy and its acceptance threshold, and the refinement/confidence rules all influenced the distribution of the meta dataset and therefore the behavior of the final learner. Because stacking trains the final model on other models' outputs, any upstream instability can propagate and sometimes amplify at the meta layer. In practical terms, the approach was hard to stabilize across variations of train/test splitting, parameter settings, and preprocessing choices.

S4.4. Pivot to the Neural Network model

For these reasons, we pivoted to a Neural Network (MLP) model as the main modelling direction. The NN approach simplified the system by replacing stacking and multiple post-hoc control mechanisms with a single end-to-end nonlinear model that learns interactions directly from the descriptor space, while still allowing explicit operating-point control through systematic threshold selection (as done in our later NN experiments). Empirically, the NN model delivered much stronger and more repeatable results than CHIRAL-META under the evaluation regimes we used for continued development, which is why it became the foundation for subsequent experimentation and reporting.

S5. Nomenclature

Table S10: Nomenclature.

Abbreviation	Description
Accuracy	Proportion of correctly classified molecules among all test molecules.
AdamW	Adaptive Moment Estimation with decoupled weight decay – optimizer used for neural network training.
AI	Artificial Intelligence – data-driven computational methods that learn patterns from data.
AUC	Area Under the Curve – generic term for the integral of a performance curve (e.g., ROC or PR).
BCE	Binary Cross-Entropy – standard loss function for binary classification.
Brier score	Calibration metric measuring the mean squared difference between predicted probabilities and true labels.
C_FN	Cost assigned to a false negative in cost-sensitive threshold optimization.
C_FP	Cost assigned to a false positive in cost-sensitive threshold optimization.
COD	Crystallographic Open Database – open-access database of small-molecule and inorganic crystal structures.
CPU	Central Processing Unit – main processor used for data preprocessing and classical machine learning tasks.
CSD	Cambridge Structural Database – curated database of organic and organometallic crystal structures.
CSP	Crystal Structure Prediction – computational methods for predicting crystal structures from molecular information.
DET	Detection Error Tradeoff – curve showing false positive rate versus false negative rate.
F1	Harmonic mean of precision and recall for a given class.
FN	False Negatives – chiral crystals incorrectly predicted as achiral.
FP	False Positives – achiral crystals incorrectly predicted as chiral.
FPR	False Positive Rate – proportion of achiral crystals incorrectly classified as chiral among all actual achiral crystals.
GPU	Graphics Processing Unit – hardware accelerator used for neural network training and SHAP computation.
J	Youden's J statistic – defined as sensitivity + specificity − 1, used for threshold optimization.
K	Number of selected descriptors retained after feature selection.
K-best	Mutual-information-based feature selection retaining the K most informative descriptors.
Macro-F1	Unweighted average F1 scores for chiral and achiral classes.
MI	Mutual Information – statistical criterion used for feature selection (K-best).
ML	Machine Learning – subset of AI using statistical models trained on data to make predictions.
MLP	Multilayer Perceptron – feed-forward neural network with one or more hidden layers.
Mordred	Python package for high-throughput calculation of thousands of molecular descriptors.

NPV	Negative predictive value; the proportion of negative predictions that are correct. Depends on class prevalence.
NVMe	Non-Volatile Memory Express – high-performance storage interface for SSD devices.
PDF	Pair Distribution Function – function describing probability of finding atom pairs at a given distance.
PDFitc	Online AI-based suite for PDF analysis, structure and space-group mining from pair distribution functions.
PDFs	Pair Distribution Functions – collection of PDF curves derived from diffraction data.
PPV	Positive predictive value; the proportion of positive predictions that are correct. Depends on class prevalence.
PR	Precision–Recall – curve of precision vs. recall across decision thresholds.
PR-AUC	Area under the Precision–Recall curve – focuses on performance for the positive (chiral) class.
RAM	Random Access Memory – system memory used for data storage during computation.
RDKit	Open-source cheminformatics library used here for molecular handling and descriptor calculation.
Refcode	Unique identifier of a crystallographic entry in the structural database (CSD refcode).
ReLU	Rectified Linear Unit – nonlinear activation function used in neural networks.
RF	Random Forest – ensemble of decision trees trained on bootstrapped samples and random feature subsets.
R-factor	Crystallographic residual factor quantifying agreement between observed and calculated diffraction intensities.
ROC	Receiver Operating Characteristic – curve of true positive rate vs. false positive rate across thresholds.
ROC-AUC	Area under the ROC curve – threshold-independent measure of class separability.
SHAP	SHapley Additive exPlanations – game-theoretic framework assigning feature-wise contributions to predictions.
SMILES	Simplified Molecular Input Line Entry System – text string encoding a molecule’s structure.
SSD	Solid-State Drive – high-speed storage device used for dataset and model storage.
TN	True Negatives – achiral crystals correctly predicted as achiral.
TNR	True Negative Rate (Specificity) – proportion of achiral crystals correctly identified.
TP	True Positives – chiral crystals correctly predicted as chiral.
TPR	True Positive Rate (Recall/Sensitivity) – proportion of chiral crystals correctly identified.
t-SNE	t-distributed Stochastic Neighbor Embedding – nonlinear dimensionality-reduction technique for visualization.
VRAM	Video Random Access Memory – GPU memory used for model training and SHAP computation.
